# Genetic Differentiation of *Abies alba* Outside Its Main Range Under Warm Meso‐ and Sub‐Mediterranean Conditions in Italy and Switzerland

**DOI:** 10.1002/ece3.70909

**Published:** 2025-02-02

**Authors:** Sevil Coşgun, Jérémy Gauthier, Giuliano Bonanomi, Gabriele Carraro, Paolo Cherubini, Marco Conedera, Erika Gobet, Maria‐Chiara Manetti, Gianluigi Mazza, Christoph Schwörer, Christoph Sperisen, Nadir Alvarez, Felix Gugerli, Willy Tinner

**Affiliations:** ^1^ Institute of Plant Sciences University of Bern Bern Switzerland; ^2^ Oeschger Centre for Climate Change Research University of Bern Bern Switzerland; ^3^ Swiss Federal Institute for Forest, Snow and Landscape Research WSL Birmensdorf Switzerland; ^4^ State Museum of Natural Sciences Lausanne Switzerland; ^5^ Department of Genetics and Evolution University of Geneva Geneva Switzerland; ^6^ Department of Agricultural Sciences University of Naples Federico II Portici Italy; ^7^ Dionea Locarno Switzerland; ^8^ Faculty of Forestry University of British Columbia Vancouver British Columbia Canada; ^9^ Swiss Federal Institute for Forest, Snow and Landscape Research WSL Cadenazzo Switzerland; ^10^ CREA Research Centre for Forestry and Wood Arezzo Italy

**Keywords:** *Abies alba*, climate change, demographic history, population genetics, silver fir

## Abstract

*Abies alba* is an important European tree species currently mostly found at cool and humid sites in the montane zone. In the past, it grew under markedly warmer and drier climates during the Eemian and mid‐Holocene, and cryptic Mediterranean populations confirm the species' capacity to grow under warm, summer‐dry conditions. However, it is unknown if warm‐loving Mediterranean occurrences are related to specific genetic properties (e.g., subspecies or ecotypes). Investigating the genetics of cryptic warm‐loving populations is crucial for a better understanding of past and future population dynamics of *A. alba* . We genotyped 478 *A. alba* samples at 174 single‐nucleotide polymorphisms (SNP), covering a broad latitudinal range from Southern Italy to Switzerland while accounting for local altitudinal gradients, and combined these newly introduced genotypes with those of other European *Abies* populations from the literature. Population genetic structure analyses grouped the warm‐loving meso‐ and sub‐Mediterranean populations into the same genetic cluster as the mountain populations of each region. The occurrence of three genetic clusters from Northern to Southern Italy is in line with the glacial refugia history. The inferred evolutionary and demographic history suggests a northward expansion of *A. alba* after glaciation, as well as a trans‐Adriatic gene flow between Balkan and Southern Italian populations. Collectively, the combined genotypic data from individuals across the species' range demonstrate that cryptic Mediterranean populations of *A. alba* align with the local and large‐scale genetic structure of populations from its main range, suggesting that the species is able to thrive in a warmer and drier environmental range than hitherto anticipated. This finding implies that it is unneeded to postulate extinct subspecies or ecotypes to explain the occurrence of meso‐ and sub‐Mediterranean Eemian or mid‐Holocene silver fir forests, with important implications for future *A. alba* population dynamics.

## Graphical abstract

By combining with individuals from across the species' range, cryptic Mediterranean populations of *A. alba* were found to align with the local and large‐scale genetic structure of populations from its main range, suggesting that the species is able to thrive in a warmer and drier environmental range than in its supposed climatic niche. This finding implies that no (extinct) subspecies or ecotypes are needed to explain the occurrence of meso‐ and sub‐Mediterranean Eemian or mid‐Holocene silver fir forests, with important implications for future *A. alba* dynamics.

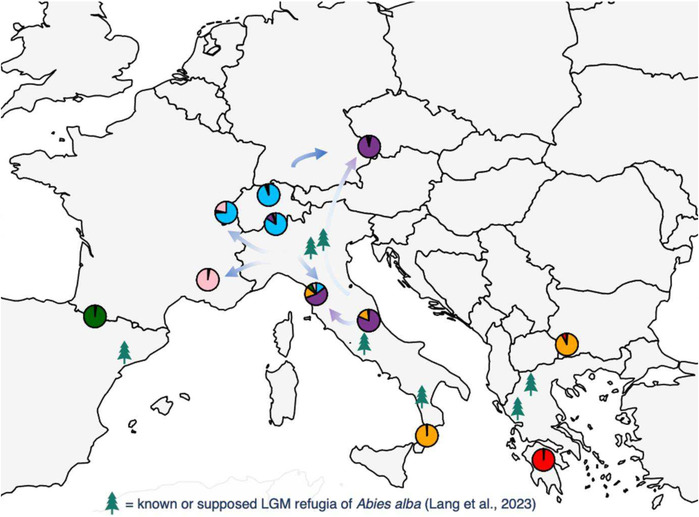

## 1 Introduction

Global change may cause significant shifts in European vegetation (Rey et al. 2020). For instance, while cold‐adapted and drought‐sensitive species are expected to be disadvantaged by changing temperature and precipitation in the near future (Fyllas and Troumbis 2009; Hanewinkel et al. 2013; Prentice, Sykes, and Cramer 1991), forest declines due to warming‐linked droughts are already reported from dry ecosystems (Camarero et al. 2011; Cherubini, Battipaglia, and Innes 2021), even for drought‐resistant species such as *Quercus ilex* (Alderotti et al. 2023).

Silver fir ( *Abies alba* ) represents an interesting case with a marked difference between current and past realized climatic niche, indicating that the species has a greater potential to cope with climate change than expected (Tinner et al. 2013; Vitasse et al. 2019; Zagwijn 1996). Dynamic, process‐based modeling results also suggest a future broader potential in Europe under global warming than so far assumed (Henne et al. 2013, 2015; Ruosch et al. 2016; Schwörer, Henne, and Tinner 2014). Together such findings raise important questions about the genetic aspects underlying its tolerance to warm climatic conditions.

A notable example of possible broad climate tolerance of the species was found in Tuscany. Several studies (e.g., Cortini Pedrotti 1967; Giacobbe 1949, 1950) reported enigmatic presences of spontaneously regenerating *A. alba* stands at very low elevations reaching the Mediterranean Sea shore, under atypical climatic conditions for the species. At the only ecologically studied site Varramista (Cortini Pedrotti 1967), such stands of unknown origin were planted at the beginning of the 19th century and thrive in competition with evergreen *Quercus ilex* at a mean July temperature of 24.5°C, exceeding the upper temperature limit in its currently known range (14°C–19°C July temperature; Mayer 1984; Tinner et al. 2013; Vitasse et al. 2019) by ca. 5°C, under dry summer conditions (97.4 mm sum of June, July, August; Walder et al. 2021). Similar conditions have been reconstructed for the Holocene Thermal Maximum (HTM), ca. 10,000–5000 years ago, when meso‐Mediterranean silver fir forests were widespread close to the shore of the Mediterranean Sea in Tuscany, in association with *Q. ilex* and other Mediterranean species, before they were destroyed by Neolithic agricultural activities (Colombaroli, Marchetto, and Tinner 2007; Henne et al. 2013; Tinner et al. 2013; Vitasse et al. 2019). Similarly, fossil records disclosed the mid‐Holocene co‐dominance of *A. alba* in competition with *Quercus pubescens, Q. petraea, Q. cerris, Ulmus* sp., and *Tilia* sp. in the sub‐Mediterranean forelands of the Southern Alps and the Northern Apennines (with July average temperatures of ca. 22°C–24°C), before the species was extirpated by anthropogenic burning and excessive browsing (Gobet et al. 2000; Tinner et al. 2013; Vescovi, Kaltenrieder, and Tinner 2010). The extant range of *A. alba* contrasts not only paleoecological evidence from the HTM but also from the Eemian, 130,000–115,000 years ago, with temperatures at least 1°C–2°C higher than today (Beaudouin et al. 2005; Kaspar et al. 2005; Zagwijn 1996). The palaeoecologically inferred warm edge of the niche might be explained by the speciation of *A. alba* , given that the species evolved in Europe during the Miocene (Badenian or Langhian), ca. 16–15 million years ago, in competition with subtropical evergreen broadleaved trees (e.g., *Phoebe*, *Laurus*, *Symplocos*, *Toddalia, Mastixia, Ficus*, *Tetrastigma*, *Buxus, Ceratonia, Olea*, evergreen *Quercus* and palms), when climatic conditions were at least 4°C–5°C warmer than today (Mai 1995).

Although some earlier studies reported lowland presences of *A. alba* in Tuscany (Giacobbe 1949, 1950), the only meso‐Mediterranean forest studied in detail so far was Varramista in Tuscany. Hence, the ecological and environmental evidence from the unique site (Cortini Pedrotti 1967; Walder et al. 2021) remained elusive. Systematic field surveys have led to the discovery of two new cryptic meso‐Mediterranean stands at Poggio Antico and Tavernelle in the same region. Moreover, we also discovered two cryptic sub‐Mediterranean stands at Corcapolo and Arcegno in Ticino, Switzerland. As cryptic populations, we consider the rare occurrences growing outside the known range or climatic niche of *A. alba* . These stands have been so far ignored or were barely considered in the literature, because the incidence of lowland sub‐ and meso‐Mediterranean *A. alba* is extremely rare today (Birks and Tinner 2016). Their presence not only confirms the palaeoecologically inferred potential of *A. alba* to grow under Mediterranean conditions but also provides the unique opportunity to examine the genetic divergence of warm‐loving populations and to explore the population genetic structure and evolutionary history of the species with a new perspective.

The population structure, genetic variation, and demographic history of *A. alba* in Europe have been investigated by several researchers (Csilléry, Buchmann, and Fady 2020; Csilléry, Ovaskainen, et al. 2020; GENTREE 2020; Konôpková et al. 2019; Liepelt et al. 2009, 2010; Martínez‐Sancho et al. 2021; Mosca, González‐Martínez, and Neale 2014; Parducci et al. 1996; Vendramin et al. 1999) across its known geographic range. However, cryptic populations from low‐elevation meso‐ and sub‐Mediterranean areas have remained unexplored. The genetic diversity of *A. alba* and the spatial distribution of its genetic lineages are mostly shaped by the postglacial recolonization history of the species (Bosela et al. 2016), similar to the other European trees (Hewitt 1999; Petit et al. 2003). We may, therefore, expect these lowland populations align with the genetic structure of the nearby mountain populations from the region, provided they do not represent plantations established from distinct origins with different climates.

In this study, we focused on the genetic composition of these cryptic meso‐Mediterranean and sub‐Mediterranean *A. alba* populations living in small and isolated stands under untypically warm and dry climatic conditions. By genotyping single‐nucleotide polymorphisms (SNPs) of samples across a wide latitudinal and altitudinal range from Southern Switzerland to Southern Italy, as well as from representative stands of Central Europe and the Carpathian/Balkan, and by comparing these genotypes with those from other European populations taken from the literature, we aim to provide the first comprehensive genetic study including lowland meso‐Mediterranean and sub‐Mediterranean *A. alba* stands to test if these cryptic stands possess a distinct evolutionary origin, as opposed to their neighboring stands from higher altitudes and to the regional gene pool.

## 2 Material and Methods

### 2.1 Study Species

*Abies alba* has a spatial range between 40° N and 52° N and from 5° E to 27° E, with isolated occurrences extending the western limit to 1° W and the southern limit to 38° N (Wolf 2003). As such, it has the most widespread distribution range among the European and Mediterranean firs (Caudullo and Tinner 2016; López‐Tirado et al. 2024). The species also covers a broad altitudinal range from around 40 m a.s.l. in Tuscany (Walder et al. 2021) to about 2900 m a.s.l. in the Pirin Mountains in Bulgaria (Tashev and Tsavkov 2014; Wolf 2003). Within this wide distribution, occurrences are concentrated at intermediate to high elevations (500–2000 m a.s.l.; Mauri, de Rigo, and Caudullo 2016). *Abies alba* is the tallest European tree species (> 60 m) and may reach 500–600 years of age under favorable conditions (Leuschner and Ellenberg 2017; Wolf 2003). It often occurs in mixed stands with *Fagus sylvatica* or *Picea abies* but may appear also in association with *Pinus sylvestris, Quercus robur, Acer* spp. or *Carpinus betulus* (Lang et al. 2023). Even though *A. alba* is known to be tolerant to different environmental conditions, such as warm or cold and light or shade, it is sensitive to late frost, low water availability, and disturbances such as repeated forest fires and excessive browsing by ungulates (Macias et al. 2006; Tinner et al. 2013; Wolf 2003).

### 2.2 Study Design and Sampling

To assess whether there are other cryptic stands of *A. alba* than Varramista in Tuscany, we undertook field surveys with the regional forestry agency, resulting in so far unknown lowland stands at Tavernelle and Poggio Antico. Similarly, surveys in Ticino led to the discovery of Arcegno and Corcapolo. Although the aforementioned lowland stands in Tuscany have a history of plantation dating back to the early 20th century from unknown geographical origins, with natural regeneration of planted trees and no active silvicultural intervention they can be considered naturalized, with exception of the recently planted and currently managed site at Pigelleto (Cortini Pedrotti 1967; Mazza et al. under review).

*Abies alba* populations were sampled in stands originating through natural regeneration or plantation across a wide latitudinal range spanning from Serra San Bruno in Southern Italy to Ticino in Southern Switzerland, following local altitudinal gradients (Table 1 and Table S2). Needle samples from Cantons of Bern and Grisons were also collected to represent Western and Eastern Swiss populations, suggested to be genetically differentiated. Samples from the Black Forest and the Carpathian Mountains were added to the sample set to represent Central Balkan/Carpathian European populations. We collected needles from around 20 adult or young trees per population, respecting at least 20 m of distance between sampled specimens to minimize the risk of sampling closely related trees. For smaller stands such as Arcegno (Ti1), which consisted of only two mature trees and a few seedlings and saplings, the number of specimens and the distance between the sampled trees were reduced (Table 1). In addition to the samples focused on in this study, specimens from Varramista collected by Walder et al. (2021) and specimens from a planted and managed forest stand next to natural regeneration in Pigelleto were collected to check their origins. Finally, to represent other European *A. alba* lineages and one *Abies cephalonica* population from Greece, data from Brousseau et al. (2016), Csilléry, Buchmann and Fady (2020), Csilléry, Ovaskainen et al. (2020), and Heer et al. (2018) were compiled for matching SNP loci (Table S1). The combined genotypic data of SNP loci matching between the specimens collected in the current study and those from the literature will be referred to as the “European set” hereafter.

**TABLE 1:** Coordinates, elevations, July temperatures, sample sizes, origin of the populations, and the stand area for studied *Abies alba* populations.

Population name	Abbreviation	Location	Coordinates (°North east)	Elevation (m a.s.l.)	July temperature^a^ (°C)	Sample size	Origin of *Abies alba*	Area of the stand (ha)^c^
Bern	Ber	Dürsrütiwald, Berne, CH	46.961, 7.777	880–920	16.95	20	Natural	L
Graubünden	Gra	Valsot, Grisons, CH	46.937, 10.481	1220–1295	14.45	20	Natural	L
Ticino1	Ti1	Arcegno, Ticino, CH	46.156, 8.742	430	21.95	10^b^	Natural	VS
Ticino2	Ti2	Corcapolo, Ticino, CH	46.173, 8.693	340	20.75	20	Natural	VS
Ticino3	Ti3	Intragna, Ticino, CH	46.181, 8.677	790–1100	18.85	18	Natural	S
Ticino4	Ti4	Intragna, Ticino, CH	46.181, 8.635	1175–1325	16.25	20	Natural	L
Ticino5	Ti5	Centovalli, Ticino, CH	46.179, 8.594	1710–1926	12.15	14	Natural	L
Ticino6	Ti6	Centovalli, Ticino, CH	46.179, 8.592	1804–1914	12.15	11	Natural	L
Ticino7	Ti7	Bosco Negro, Ticino, CH	46.187, 8.564	1380–1494	15.35	20	Natural	L
Ticino8	Ti8	Anzonico, Ticino, CH	46.433, 8.867	1175–1324	13.95	11	Natural	L
Tuscany1	Tu1	Varramista, Tuscany, IT	43.658, 10.715	33–58	24.05	20 (52^b^)	Naturalized	VS
Tuscany2	Tu2	Tavernelle, Tuscany, IT	43.546, 12.014	360–405	22.65	20	Naturalized	S
Tuscany3	Tu3	Pigelleto, Tuscany, IT	42.811, 11.658	747–836	20.65	24 (46^b^)	Natural/Plantation	S
Tuscany4	Tu4	Poggio Antico, Tuscany, IT	43.027, 11.474	521–552	22.35	20	Naturalized	VS
Tuscany5	Tu5	Abetone, Tuscany, IT	44.152, 10.679	1434–1462	15.15	20	Natural	M
Molise1	Mo1	Pescopennataro, Molise, IT	41.866, 14.294 41.851, 14.291	1249–1471	17.15	20	Unknown	L
Molise2	Mo2	Collemeluccio, Molise, IT	41.709, 14.355	834–935	19.85	20	Natural	M
Basilicata1	Ba1	Monticchio, Basilicata, IT	40.930, 15.615	665–688	22.25	9 (19^b^)	Natural/Plantation	S
Basilicata2	Ba2	Laurenzana, Basilicata, IT	40.406, 15.958	1108–1128	18.55	20	Natural	L
Basilicata3	Ba3	Terranova di Pollino, Basilicata, IT	39.966, 16.217	1413–1468	16.45	20	Natural	L
Campania	Cam	Monte Motola, Campania, IT	40.374, 15.460	995–1280	17.35	20	Natural	S
Calabria	Cal	Serra San Bruno, Calabria, IT	38.557, 16.314 38.547, 16.289	823–1071	20.55	20	Plantation	M
Black Forest	BF	Nagold, Baden‐Württemberg, GER	approx.48.54, 8.73		—	14	Natural	—
Romania	Rom	Gura Humorului, Bukovina, RO	approx. 47.51, 25.88		—	9	Unknown	—

^a^ Climatic data were obtained through CHELSA V 2.1 for 1981–2010 (Karger et al. 2017, 2018).

^b^ Sample sizes, including the samples removed following the STRUCTURE analysis.

^c^ L, Large (> 500 ha); M, Medium (100–500 ha); S, Small (< 100 ha); VS, Very small (< 10 ha).

### 2.3 DNA Extraction and SNP Genotyping

DNA extractions were performed with ~25 mg lyophilized and ground needle tissue per sample using sbeadex maxi plant kit (LGC Genomics, Berlin, Germany) according to the manufacturer's protocol. DNA quality and quantity were assessed with a NanoDrop spectrometer (Thermo Fisher Scientific). A total of 478 *A. alba* DNA samples were genotyped at 174 putatively neutral SNPs using KASP arrays by LGC Genomics (Berlin, Germany), as detailed in Appendix S2 and S3. These SNPs, described by Roschanski et al. (2016), are mainly located in coding regions and predominantly show synonymous mutations.

### 2.4 Population Genetic Structure

Genetic structure was assessed using the Bayesian clustering algorithm implemented in the software STRUCTURE v.2.3.4 (Falush, Stephens, and Pritchard 2003, 2007; Hubisz et al. 2009; Pritchard, Stephens, and Donnelly 2000). A separate STRUCTURE analysis was performed with the European set, comprising 112 SNPs shared among the specimens from this study and those of the literature (Appendix S2 and S3). Principal component analyses (PCA) and hierarchical clustering were carried out with the R packages *adegenet* v.2.1.8 (Jombart 2008; Jombart and Ahmed 2011; Jombart, Devillard, and Balloux 2010) and *dendextend* 1.17.1 (Galili 2015), respectively.

### 2.5 Genetic Diversity

Observed heterozygosity (*H*_o_), expected heterozygosity (*H*_e_), mean allelic richness (*A*_r_), the inbreeding coefficient (*F*_is_), and the fixation index (*F*_st_) were estimated for each sampling site using *adegenet* v.2.1.8 and *hierfstat* (Goudet 2005). Populations were also tested for Hardy–Weinberg equilibrium with χ^2^ statistics with 1000 permutations using *pegas* (Paradis 2010). Diversity estimates were tested for latitudinal trends using linear regression implemented in SPSS 28.0. Isolation by distance was tested for the European set (removing Pyrenees and Greece because of their remote locations) in a matrix correlation of Spearman's Rho, using geographic distances calculated by *geosphere* 1.5–1.8 R package (Hijmans et al. 2022) and linearized genetic distances (*F*_st_/(1 − *F*_st_); Rousset 2000). Further details are given in Appendix S2 and S3.

### 2.6 Evolutionary Relationships

Evolutionary relationships among populations in the European set were investigated using TreeMix v.1.13 (Pickrell and Pritchard 2012). This method creates a maximum likelihood tree through allele frequencies, adds migration for populations with multiple parental populations, identifies admixture among sets of three populations (f3‐statistics), and determines populations that are poorly fitting to this model by estimating residuals (Pickrell and Pritchard 2012; further details are given in Appendix S3).

### 2.7 Demographic History Analysis

For inferring the demographic history of *A. alba*, approximate Bayesian computation was carried out using DIYABC‐RF (Collin et al. 2021; Cornuet et al. 2014) based on the European set. To simplify the procedure and to have a uniform sample size, ~100 samples were selected randomly from each genetic cluster. These clusters were defined based on STRUCTURE results, except for Switzerland. Swiss samples were divided into three groups, Southern (Ticino), Western, and Central/Eastern Switzerland, to be able to check for suggested migration routes. Priors for the divergence times of Central/Eastern Switzerland, Tuscany, and Bavaria populations were determined based on the Holocene expansion inferred from paleoecological records in the literature. We employed “ghost populations” to represent putative lineages that have not been sampled in this study. Further details can be found in Appendix S1.

## 3 Results

### 3.1 Population Genetic Structure

**FIGURE 1:**
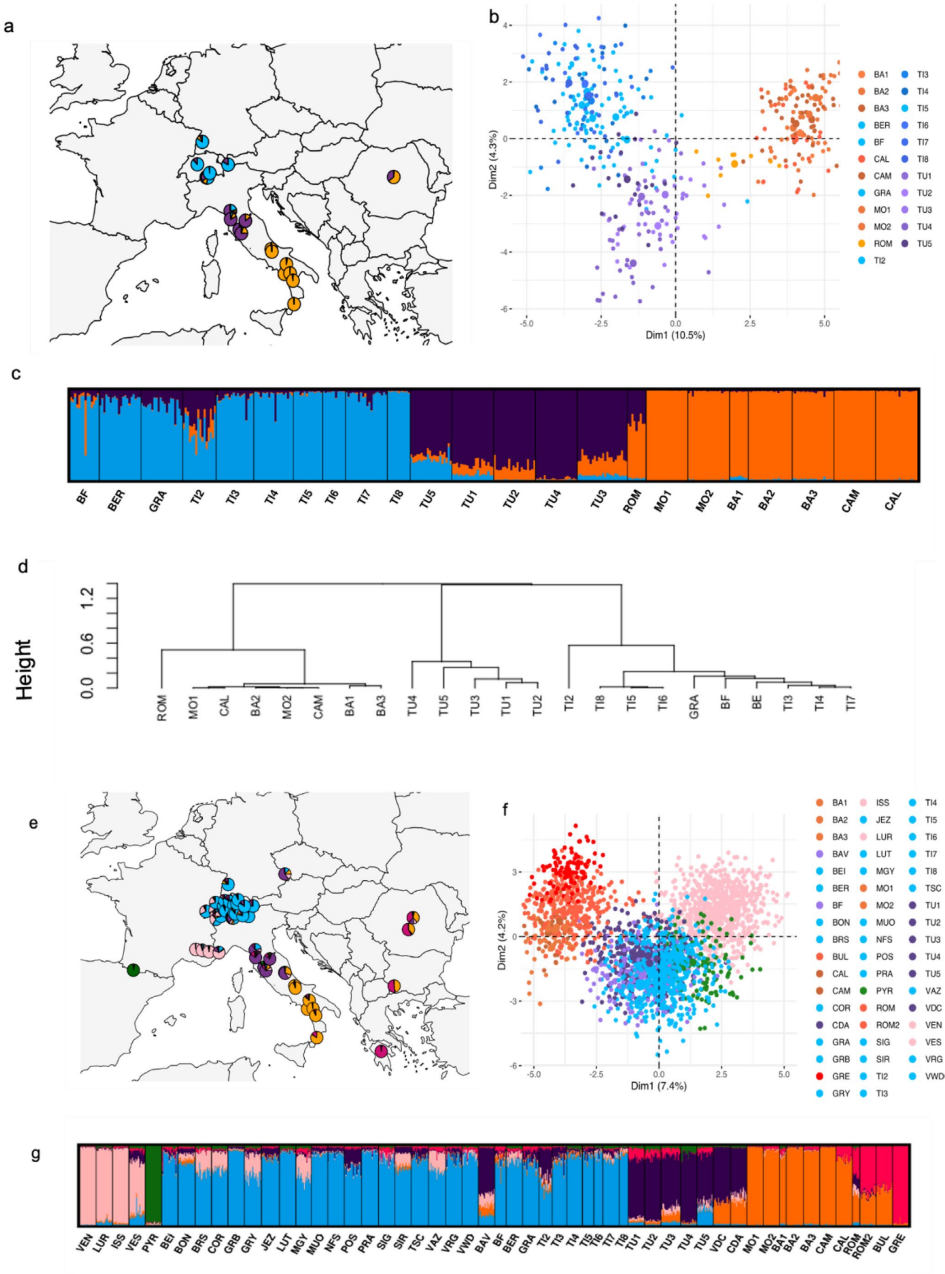
Genetic structure of *Abies alba*. STRUCTURE assignment for population samples from this study (*K* = 3) (a, c) and the European set (*K* = 6; with literature data, Greece population represents *Abies cephalonica*) (e, g). Principal component analysis with populations sampled in this study (b) and with the European set (f). Hierarchical clustering of the samples collected in this study based on STRUCTURE analysis (*K* = 3) (d).

STRUCTURE results indicated spatial clustering across Italy and Switzerland (Figure S1). The stand at Arcegno (Ti1) showed a clear separation from the neighboring stands, most probably due to the sampling of closely related trees; accordingly, it was excluded for further analyses. Similarly, a part of the Basilicata 1 (Ba1) samples was removed prior to statistical analyses. Ba1 consists of samples from the shore of Lago di Monticchio and the nearby Monte Vulture peak. Monte Vulture specimens showed a significantly different genetic structure than other Basilicata and Southern Italian samples, including those from low elevations at Lago di Monticchio. This difference was attributed to the reforestation of Monte Vulture in the 20th century (Zampino, Falconeri, and Navazio 2017) with non‐local seed sources. Therefore, only low‐elevation lake shore samples were kept for further analyses. In addition, the Varramista (Tu1) samples collected by Walder et al. (2021) and the Pigelleto (Tu3) samples from the forest management site were removed to avoid duplicate representation. Following the removal of these sub‐populations, STRUCTURE analysis was performed again (Figure 1a,c and Figure S2b).

STRUCTURE analyses assuming two genetic clusters (*K* = 2) divided the populations into Southern Italy/Balkan (including the Carpathians) and the rest (i.e., Central Italy, Northern Italy/Switzerland/Germany; Figure S2) for both, this study's samples and the European set. Tuscan populations represented an admixture of Northern Italian/Swiss and Southern Italian populations. In accordance, estimates of genetic differentiation (*F*_st_) were lower among Swiss and Tuscan populations than those from Southern Italy (Appendix S3—Nei's Fst). With *K* = 3, the separation of Tuscan populations became apparent (Figure 1a,c,d) in line with PCA (Figure 1e). Poggio Antico (Tu4) showed almost no signs of admixture. For the samples collected in this study, *K* = 4 was determined to describe best the genetic structure (Figure S5b) and put Tu4 forward as a separate genetic cluster. Further *K* values revealed more admixture among the Tuscan and Ticino populations with no clear geographical pattern. STRUCTURE analysis of the European set revealed a clustering consistent with that of the samples from this study. However, Tu4 was not identified as a separate cluster but consistently showed a low level of admixture in contrast to other Tuscan populations. Additional clusters were formed by populations of France (*K* = 4), Pyrenees (*K* = 5), and *Abies cephalonica* in Greece (*K* = 6) (Figure S2).

The meso‐Mediterranean populations in Tuscany (Varramista‐Tu1 and Tavernelle‐Tu2) did not show any distinct structure and were thus assigned to the same genetic cluster as other Tuscan populations, with admixed ancestry from Northern and Southern Italy sources. Combining these genotypes with the literature data (Figure 1e,g) in the European set shows that our Tuscan populations broadly correspond to the lineage of the Central Apennine populations described in Brousseau et al. (2016).

### 3.2 Genetic Diversity

**FIGURE 2:**
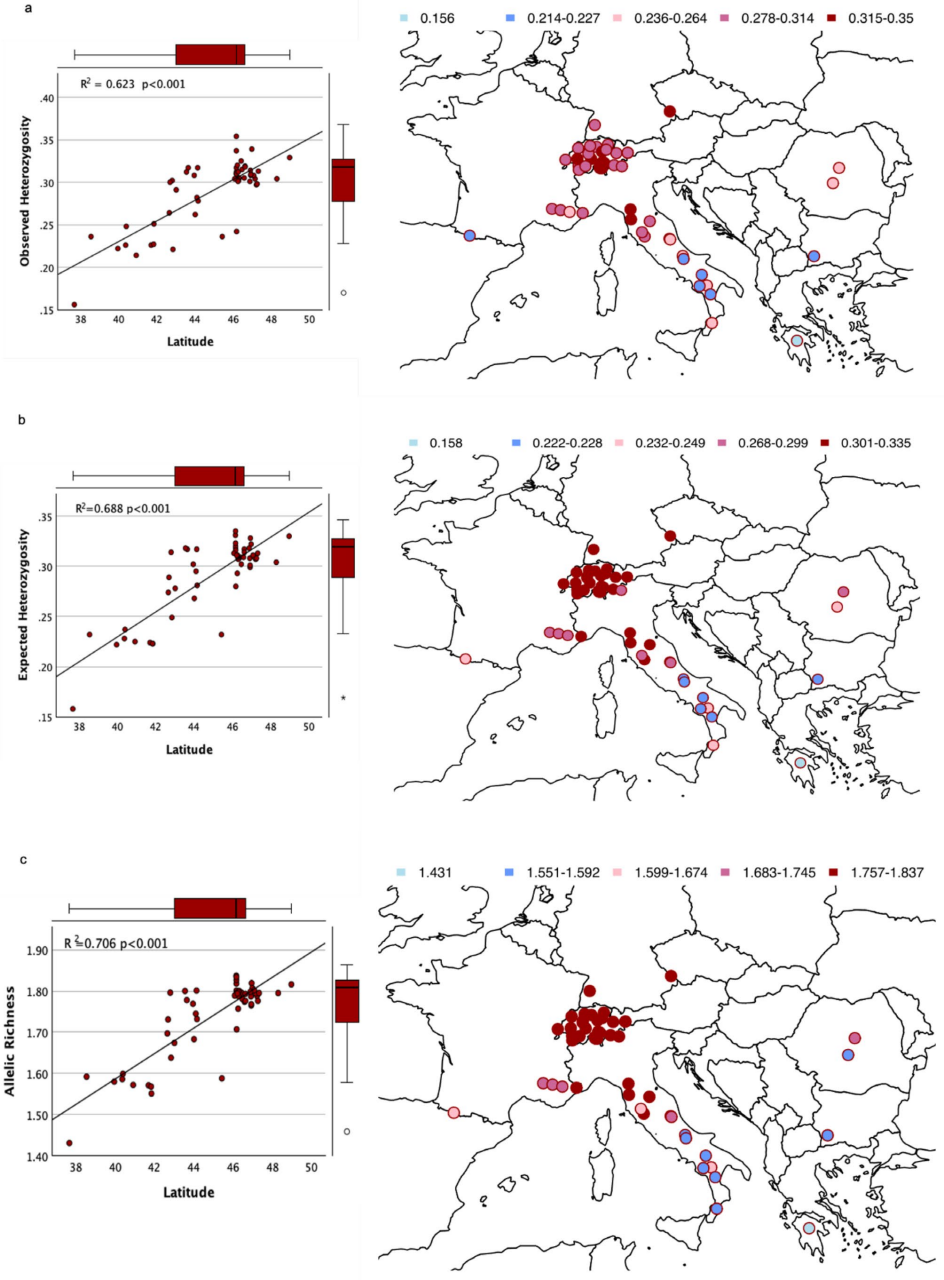
Observed heterozygosity (*H*_o_; a), expected heterozygosity (*H*_e_; b), and allelic richness (*A*_r_; c) for the European set of *Abies alba* with linear regressions of diversity measures on latitude (with literature data; the Greece population represents *Abies cephalonica*); *R* ^2^ values and levels of significance are given in the insets.

Observed (*H*_o_), expected heterozygosity (*H*_e_) and allelic richness (*A*_r_) were estimated for the samples from this study and the European set, and all three parameters showed prominent and statistically significant (*p* < 0.001) trends of an increase with northern latitude in both sets of populations (Figure 2 and Figure S3). For the European set, diversity indices were higher in Northern populations in general, but with the highest allelic richness reaching as far south as ca. 42° N (i.e., Central Italy). Southern Italy, the Pyrenees, and the Balkans had the lowest diversity indices (*H*_o_ = 0.214–0.251, 0.221, and 0.227–0.247, *A*_r_ = 1.551–1.599, 1.638, and 1.568–1.707 for Southern Italy, the Pyrenees, and the Balkans, respectively), excluding the Greek population, which belongs to the closely related species *A. cephalonica*. Similar trends were observed for the samples collected in this study, with a decreasing trend from North to South (Figure S3 and Table S3). Isolation by distance was also statistically significant for the European set (Mantel *r* = 0.8083, *p* = 0.001; Figure S4).

### 3.3 Evolutionary Relationships

**FIGURE 3:**
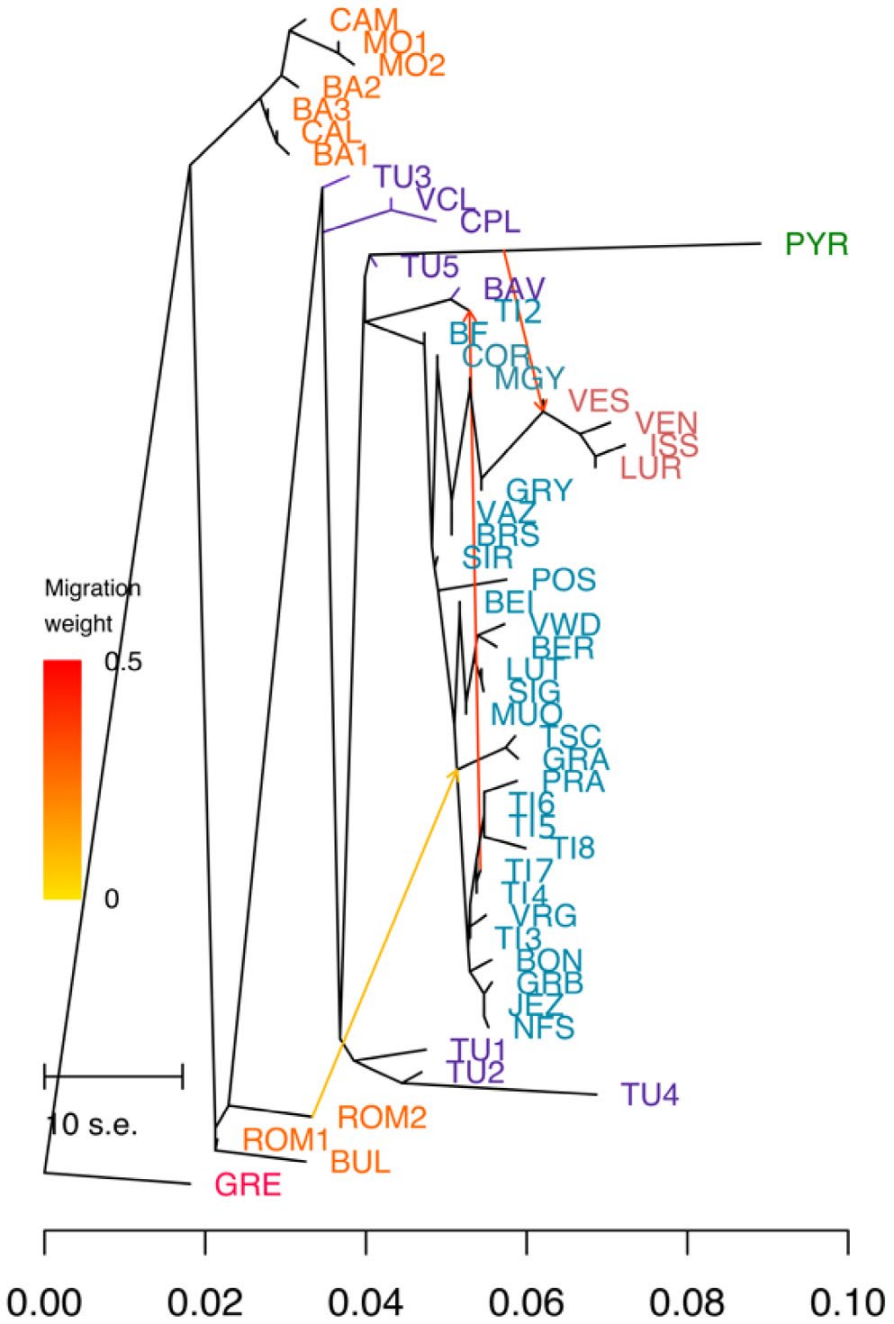
Maximum likelihood tree for the European set of *Abies alba* (with literature data; GRE represents *Abies cephalonica*).

The tree obtained by TreeMix for the European set revealed that the Southern Italian and Balkan clusters branch off first, pointing to the oldest divergence (Figure 3). The Central Italian sites Valle della Corte (Vdc) and Colle dell'Abete (Cda) formed another clade, while Pigelleto (Tu3) and Abetone (Tu5) did not group with other Tuscan sites. Indeed, Northern Italian/Swiss, Tuscan, and German populations showed a scattered and irregular clade in general, most probably due to high admixture between populations. On the other hand, the Pyrenees (Pyr) and Tuscan Poggio Antico (Tu4) populations showed a higher genetic drift. Three migrations [Figure S6; from Pyr to Vesubie (Ves): 36%, from Bosco Negro (Ti7) to Corcapolo (Ti2): 40%, and from Romania (Rom) to Tschlin (Tsc) and Grisons (Gra): 24%] were estimated by optM (Fitak 2021). With this model, the maximum likelihood tree explained 91.12% of the total variation. However, the presence of residuals above zero indicates that the model could not wholly explain the evolutionary history of the samples (Figure S7). Additionally, the f3 test revealed some admixture between populations (Appendix S3—f3). The detected admixtures were mostly within Northern Italian/Swiss stands, while some significant *Z*‐scores were obtained for the admixture between French, Swiss, and German populations. On the other hand, admixture between Southern Italy and the Balkans was also estimated (Calabria > Basilicata 1 & Romania 1, *Z*‐score = −3.38779).

### 3.4 Demographic Analysis

Due to the complex postglacial spread of *A. alba* , estimating the demographic history over a single simulation was unfeasible. Therefore, a stepwise procedure was followed to determine the best scenario for each population in the European set (see Appendix S1 for details). In the first simulation, we tested four scenarios for French and Tuscan populations: (I) the establishment of French populations directly from ghost refugia, (II) admixture with the Pyrenees, (III) the establishment of Tuscan populations directly from the Central Apennines, and (IV) admixture with a ghost population. The random forest algorithm selected the recolonization of France from ghost refugia and admixture between ghost refugia and the Central Apennines for Tuscan populations (posterior probability *P* = 0.623) as the most probable scenario. Considering the admixture of Southern Italian and Balkan populations as suggested by TreeMix, we tested three scenarios for Southern Italy: (I) recolonization from Southern Italian refugia, (II) admixture with Balkan *A. alba* , and (III) admixture with *A. cephalonica*. DIYABC‐RF supported the Balkan *A. alba* admixture (*P* = 0.677). Further simulations were run for Bavaria, where DIYABC‐RF selected the contribution of both the Central Apennines and Central/Eastern Switzerland over direct migration from the Central Apennines (*P* = 0.878).

Considering several potentially involved refugia and migration routes, the final simulation for Switzerland was run with six scenarios by combining the previous outputs into a baseline scenario: (I) Ticino and Western Switzerland were recolonized from ghost refugia and Central/Eastern Switzerland originated from both Southern and Western Switzerland, (II) Ticino was recolonized from ghost refugia, and Western Switzerland was recolonized from ghost refugia and France, and; further scenarios add migration from the Balkans to Eastern Switzerland as suggested by TreeMix and an admixture of Central Apennines in Ticino as observed in the population genetic structure. Even though the first scenario was selected by random forest, the probabilities of Scenarios I and II were quite similar (0.338 and 0.306), with an overlap in the first two axes of linear discriminant analysis (LDA). However, a further separation could be observed on the third axis, favoring Scenario I. Further details can be found in Appendix S1.

Assuming 50 years of generation time (Piotti et al. 2017), divergence/contact times t1 (Bavaria), t2 (Tuscany and Central/Eastern Switzerland), t3 (France, Western Switzerland, and Ticino), t4 (Pyrenees), t5 (Southern Italy), and t6 (all lineages from a common ancestor) were estimated as 5750 (95% CI: 1150–10,900), 8650 (95% CI: 3400–12,250), 619,250 (95% CI: 294,600–943,800), 565,450 (95% CI: 152,900–939,050), 575,200 (95% CI: 100,950–947,650), and 1,863,700 (95% CI: 1,140,450–2,445,000) years ago, respectively.

## 4 Discussion

In recent times, the ecological re‐evaluation of the publication by Cortini Pedrotti (1967) on the surprising presence of meso‐Mediterranean *A. alba* in Varramista attracted the interest of an increasing number of researchers (e.g., Lang et al. 2023; Tinner et al. 2013; Vitasse et al. 2019; Walder et al. 2021). The study of this silver fir forest underscored the ability of the species to grow and regenerate in meso‐Mediterranean lowlands in competition with Mediterranean species such as *Q. ilex* , even though Mediterranean summer drought can be a key limiting factor for *A. alba* (Walder et al. 2021). While our field survey let us discover the existence of other cryptic meso‐ and sub‐Mediterranean silver fir stands, our population genetic structure and evolutionary and demographic history analyses reveal that these lowland populations match the regional setting, documenting the capability of the species to survive under a wider climatic range than hitherto commonly anticipated.

### 4.1 The Current Distribution of Lineages

Population genetic structure analysis with the samples collected for this study combined with other European populations from the literature showed a divergence of Southern Italy, Northern Italy/Central Europe (Switzerland, Black Forest), Southern France, Pyrenean, Central Italy/Central Europe (Bavaria), and Greece (*A. cephalonica*) with *K* = 6. Based on a larger dataset of 290 SNPs and 71 populations, the GENTREE (2020) report identified five clusters: Balkan/Southern Italy, Northern Italy/Central Europe (France, Switzerland, Germany), Southern France, Pyrenean, and Central Italy/Central Europe (Austria, Czech Republic). Differing from GENTREE (2020) when including *A. cephalonica* in our European set, the Balkan *A. alba* showed a pattern of assignment not directly identical to Southern Italy but also with admixture from *A. cephalonica*. Indeed, the findings of Ziegenhagen et al. (2005) and Siskas et al. (2023) revealed some mitotypes and chlorotypes shared among these two species in the Balkans and laid out the relationship between these close relatives and their populations in detail.

So far, nothing has been known about the origin of the planted lowland populations of *A. alba* in Tuscany, impeding a thorough assessment of whether these stands are of local origin or potentially originating from other areas within the Mediterranean realm. Distant Mediterranean provenances, perhaps admixed with other drought‐resistant species such as *A. cephalonica*, would question the heat‐ and drought‐tolerant potential of extant *A. alba* populations as suggested by palaeoecological evidence (Lang et al. 2023; Tinner et al. 2013; Vitasse et al. 2019). Instead, the warm‐loving lowland populations we studied did not show a different assignment to ancestral clusters than their neighboring populations at higher and cooler elevations. More specifically, our findings indicate a local or regional origin for the Tuscan meso‐Mediterranean and the Ticino sub‐Mediterranean lowland stands, suggesting a broader climatic niche for the species than commonly assumed today. However, given the sub‐clustering of Tuscan populations with a further number of clusters, a population genetic analysis focusing on the fine‐scale structure of Apennine populations may bring further insights.

### 4.2 Refugial Areas With Reduced Genetic Diversity

Populations in or near glacial refugial areas are expected to be highly divergent due to long periods of isolation during ice ages, whereas genetic diversity in populations distant from the refugial origins tends to be comparatively low due to (repeated) founding events during recolonization (Petit et al. 2003). However, this general assumption might overlook a more complicated mechanism underlying the current diversities over refugial and colonized areas (Widmer and Lexer 2001). The study of Comps et al. (2001) suggests that bottlenecks during recolonization may lead to an irreversible loss in allelic richness, while an opposite trend in heterozygosity can be observed due to admixture.

Paleoecological studies revealed several Last Glacial Maximum (LGM) refugia for *A. alba* in Italy: Southern Italy (e.g., Allen, Watts, and Huntley 2000; Grüger 1977; Watts, Allen, and Huntley 1996), the Central Apennines (Follieri et al. 1998), the Northern Apennines (e.g., Hofstetter et al. 2006; Vescovi, Kaltenrieder, and Tinner 2010), and Northeastern Italy (e.g., Gubler et al. 2018; Kaltenrieder et al. 2010; Samartin et al. 2016). As proposed by Hewitt (2004), populations in southern peninsulas have diverged and accumulated genetic differences over many glacial cycles, while northern populations have been regionally extinct, and the area was recolonized repeatedly. As Southern Italian and Balkan populations diverged first (Figure 3), one may expect high allelic richness there. However, we found higher diversity in other refugial areas (Central and Northern Italy), and some areas which were colonized during the Holocene only (e.g., Black Forest populations) (Figure 2, Figure S3, and Table S3). Similar results can be found in the literature, such as the study of Vendramin et al. (1999), even though there are also contrasting findings with different markers, such as Piotti et al. (2017). More recently, GENTREE (2020) illustrated a very similar trend with lower diversity further south, suggesting that during glaciations, southern populations survived with small population sizes, enhancing the effects of genetic drift, hence the loss of genetic diversity. An increasing genetic differentiation toward the north has also been reported for some other European tree species (Milesi et al. 2024), and northern populations may have been more affected by gene flow between lineages from multiple refugial populations, which contributes toward an increase in genetic diversity. As seen in the population genetic structure, populations north of ca. 42° N reveal admixture from different lineages, whereas Southern Italian and Balkan populations showed a more isolated genetic composition. In addition, we detected a significant isolation by distance (IBD) trend for the geographic distribution of *A. alba* in the European set (Figure S4), with a strong positive relationship with distance, until increasing effects of genetic drift induce a scatter of values of genetic differentiation over long distances, which is typical for large‐scale sampling (Gugerli et al. 2023) and reflects increasing effects of genetic drift over larger geographic distance (Hutchison and Templeton 1999).

### 4.3 Genetic Structure Reflecting Past Legacies

The genetic structure of forest trees is strongly shaped by refugial populations during Pleistocene glacial cycles and recolonization processes (Hewitt 1999); therefore, the divergence of different *A. alba* lineages can be attributed to glacial refugia and postglacial migration. Following the LGM and the last Heinrich Event (HE 1), around 16,000 years ago, temperate species started to expand northward with a warming climate and retreating ice sheets (Hewitt 1999). In Southern Europe, *A. alba* markedly expanded during the Bølling–Allerød interstadial, 14,600–12,800 calibrated years before present (cal yr. BP), slowed or even retreated during the Younger Dryas cold period, 12,800–11,700 cal yr. BP, and continued to spread during the early and mid‐Holocene (Giesecke et al. 2017; Liepelt et al. 2009; Ruosch et al. 2016). Specifically, following the 8.2 ka event (cooling by ca. 1.5°C–2°C over Europe with reduced drought stress), *A. alba* expanded into Central Europe by virtue of a more favorable oceanic climate (Tinner and Lotter 2001).

**FIGURE 4:**
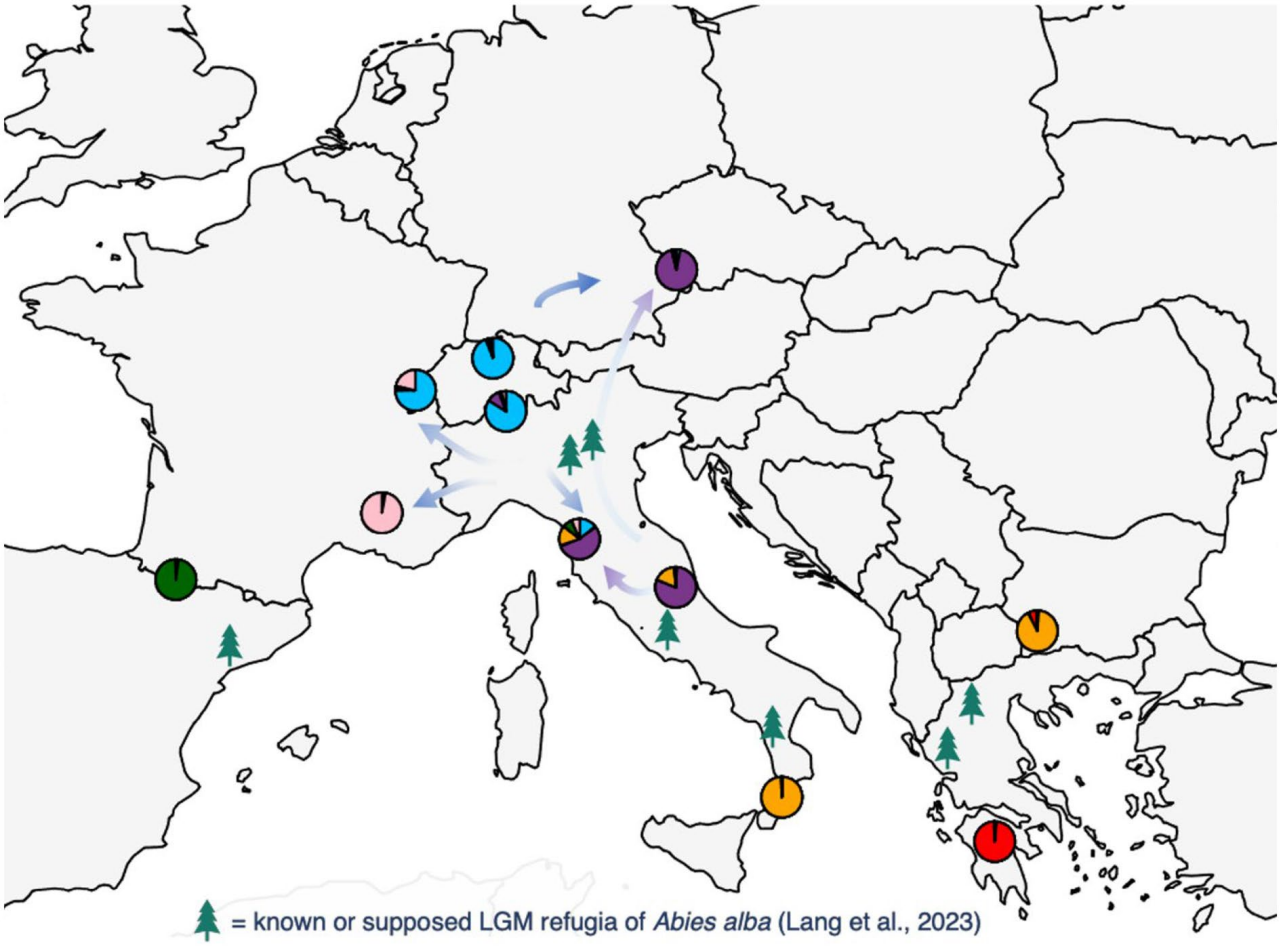
Map of the postglacial history of *Abies alba* based on population genetic structure and demographic history simulations (for the European set). Pie charts represent the STRUCTURE clusters of random samples from each population (*K* = 6). Arrows refer to putative migration routes; the Greek population represents *Abies cephalonica*.

Our demographic history analyses revealed that the Tuscan populations originated from the Central Apennines lineage with the contribution of a ghost population, which is in line with the populations' genetic structure. Paleoecological studies confirmed a glacial refugium at the Tyrrhenian side of the central Apennines (Follieri et al. 1998), which may be involved in the recolonization of *A. alba* in Tuscany. Palaeoecologically inferred Northern Italian LGM refugial populations (e.g., Kaltenrieder et al. 2010; Lang et al. 2023; Vescovi, Kaltenrieder, and Tinner 2010) might be the “ghost” in our demographic analyses and could represent the main contribution to the northward postglacial recolonization of *A. alba*. Earlier studies, such as Mayer (1973), proposed that *A. alba* expanded to the Alpine region mainly from Southern Italian refugia (Parducci et al. 1996). However, in agreement with the new palaeoecological evidence, genetic studies revealed that northward re‐immigration was only possible from Central or Northern Italy as inferred from the peculiar genetic characteristics of Southern Italian *A. alba* (Burga and Hussendörfer 2001; Konnert and Bergmann 1995; Liepelt et al. 2009; Vendramin et al. 1999). In accordance with the population genetic structure, analyses of the demographic history confirmed the recolonization of the Swiss and French Alps from Northern Italy, as suggested by Muller et al. (2007), whereas the different lineages of French and Swiss populations may be due to mid‐Pleistocene separation and indicate recolonization from different LGM refugia (Figure 4).

Concordant and discordant aspects emerged from evolutionary relationships and demographic history. TreeMix suggests a migration from the Pyrenees to France (Figure 3). The Pyrenees, together with Southern Italy, represents an isolated refugial area with a distinct gene pool (Konnert and Bergmann 1995; Vendramin et al. 1999). Indeed, Scotti‐Saintagne et al. (2021) reported an admixture with the *A. alba* gene pool of the Pyrenees for the Massive Central and Corsica but not for the Western Alps (like the French populations we employed from Brousseau et al. (2016)). However, a spread from the Pyrenees to the French Alps is rejected by approximate Bayesian computation in our analyses.

Another remarkable result from TreeMix is the local and historical influences on the genetic structure of the lowland stand at Corcapolo (Ti2) in Ticino. The population genetic structure analysis indicated that Ti2 is mainly aligned with the nearby mountain populations, with admixture from Tuscany and Southern Italy. This alignment was coupled with migration from Bosco Negro (Ti7) to Ti2 as suggested by TreeMix, indicating that the genetic structure of this lowland population is mostly shaped by local gene flow. However, the branching of Ti2 with BAV by TreeMix reveals admixture from Tuscany that contributed additional genetic diversity. TreeMix analyses also suggested another migration route from the Balkans to Eastern Switzerland (Figure 3), corroborating the findings of Burga and Hussendörfer (2001), reporting the presence of region‐specific alleles from eastern populations in some Swiss individuals, even though such a scenario was not confirmed by DIYABC‐RF in our analysis. However, the rejection of some of these scenarios could be due to different sampling densities and incomplete representation of the actual demography, as resulting from the limited number of SNPs or different markers employed in the literature.

Furthermore, TreeMix revealed admixture between populations of the Balkans and Southern Italy, in line with the findings of Piotti et al. (2017), most probably due to pollen‐mediated trans‐Adriatic gene flow, as already suggested by Liepelt, Bialozyt, and Ziegenhagen (2002). Such long‐term pollen‐based admixture likely reduced the genetic divergence between Southern Italy and the Balkan populations. In accordance, DIYABC‐RF supports an admixture event between the Southern Italian and Balkan populations around 575,200 years ago (95% CI: 100,950–947,650). The pronounced uncertainty aligns with the finding of Piotti et al. (2017), who report a connection around 237,500 years ago with a wide confidence interval (95% CI: 90,000–342,500). Given the broad range of confidence intervals, the timings should be treated carefully, pointing to the existence of an admixture zone established during the mid‐Pleistocene.

Even though our analyses reveal the mid‐Pleistocene genetic divergence of the Ticino population, the area was ice‐covered during the LGM, and it is well documented that the postglacial establishment of *A. alba* occurred around 13,500–11,500 cal yr. BP and 10,500 cal yr. BP at higher and lower elevations, respectively (Hofstetter et al. 2006). The widespread presence of meso‐ and sub‐Mediterranean stands of *A. alba* in the mid‐Holocene is evidenced by paleoecological studies in many locations and led to controversial interpretations. Tinner et al. (1999) demonstrated the co‐dominance of *A. alba* in the lowlands of Ticino (Southern Switzerland), while nearby records confirmed the importance of the species in lowland Lombardy (Northern Italy; e.g., Drescher‐Schneider and Tobolski 1985; Gobet et al. 2000; Martinelli et al. 2017) and Piedmont (Northern Italy; Finsinger and Tinner 2006) under sub‐Mediterranean conditions. Further south, Tuscany was hosting meso‐Mediterranean *A. alba* (Colombaroli, Marchetto, and Tinner 2007).

The distribution range of *Abies alba* began to contract in different areas at different times between 7000 and 2000 years BP, and warm‐tolerant Mediterranean as well as cold‐adapted treeline populations became locally extinct, narrowing the realized niche of the species (Vitasse et al. 2019). In the Late Neolithic (ca. 5000 cal yr. BP for Lago di Origlio, Lago di Muzzano and Lago di Como; Martinelli et al. 2017; Tinner et al. 1999) and during the Bronze Age (ca. 3500 cal yr. BP at Balladrum; Hofstetter et al. 2006), lowland *A. alba* populations in the Southern Alpine forelands of Northern Italy and Switzerland were significantly disturbed and then locally extirpated. Colombaroli, Marchetto, and Tinner (2007) and Drescher‐Schneider et al. (2007) also reported drastic Neolithic collapses in lowland populations around Lago di Massaciuccoli and Lago dell'Accesa at ca. 6000 cal yr. BP in Tuscany in Central Italy. While an earlier interpretation attributed the Holocene extinction of the sub‐Mediterranean populations to climate change and human impact (Wick and Möhl 2006), recent research suggested that the slash‐and‐burn and grazing activities led to major changes in vegetation in Southern Switzerland and Northern Italy (Colombaroli and Tinner 2013; Martinelli et al. 2017). Moreover, Wick and Möhl (2006) proposed that the low genetic variability of *A. alba* made the species susceptible to Holocene climatic change; however, the ancient DNA (aDNA) study by Schmid et al. (2017) disclosed the recovery of genetic variation in Ticino populations following the mid‐Holocene decline between 6500 and 6200 cal yr. BP. This first marked decline in *A. alba* populations coincides not only with a dry period according to data derived from the lake level (Magny et al. 2012) and speleothems data (mineral deposits in caves; Regattieri et al. 2019) but also with an increase in slash‐and‐burn activities (Martinelli et al. 2017). Even though we cannot comment on the true driver(s) of the local extinction from a genetic perspective, the findings of Schmid et al. (2017) and our own data on extant cryptic stands show no sign of genetic impoverishment of sub‐Mediterranean Ticino populations of *A. alba*. The ability of *A. alba* lineages of Northern and Central Italy to survive under Mediterranean temperatures at the sites where cryptic populations were found supports the reconstruction of a broader mid‐Holocene climatic niche. However, further studies, including aDNA and population genetic studies with a larger SNP set, are required to corroborate that the extant stands and the mid‐Holocene lowland sub‐Mediterranean and meso‐Mediterranean populations are genetically linked or even show genomic signatures of adaptation related to the respective climatic drivers (Schwörer et al. 2022).

Realized niches of species are determined by not only climatic conditions but also competition, facilitation, physical barriers to dispersal, and anthropogenic impact, suggesting that focusing solely on current conditions may underestimate species' capacity for growth under global warming (Zhao and Wang 2023). Studies have been carried out to estimate the (masked) potential of important tree species in Europe in drier and warmer climates. Results of common garden experiments and provenance trials indicate that *A. alba* populations from warm and dry habitats show a higher drought tolerance than those from humid areas, presumably reflecting local adaptation (e.g., Csilléry, Buchmann, and Fady 2020; Mihai et al. 2021). Regarding *A. alba* , evidence from very dry areas of Europe, such as the Hungarian Plain, shows that if planted, *A. alba* can thrive surprisingly well under conditions clearly outside its current range and supposed climatic niche (Mátyás et al. 2021). Moreover, the observed spontaneous regeneration of Southern Italian, Central Italian, and Northern Italian/Southern Swiss *A. alba* lineages in a wide altitudinal and climatic range, partly in competition with Mediterranean species such as *Q. ilex*, reveals their true capability to grow under warmer and drier conditions. However, the growth of the species is still limited by environmental conditions such as severe summer drought or insufficient water holding capacity (Walder et al. 2021), making further studies needed to verify their actual performance under these warm and summer‐dry Mediterranean conditions.

## 5 Conclusion

Cryptic lowland populations of *A. alba* in Tuscany and Ticino mirror the regional genetic structure with no distinct evolutionary history, suggesting that the species is able to thrive in a warmer and drier environmental range than its currently realized climatic niche. These findings support the attribution of Holocene lowland population declines to anthropogenic pressure and indicate that *A. alba* may be a key species for sustaining forest ecosystem services in a warmer world under ongoing climate change.

## Author Contributions

**Sevil Coşgun:** conceptualization (equal), investigation (lead), methodology (lead), visualization (lead), writing – original draft (lead). **Jérémy Gauthier:** conceptualization (equal), investigation (equal), methodology (equal), writing – review and editing (lead). **Giuliano Bonanomi:** investigation (equal), methodology (supporting), writing – review and editing (supporting). **Gabriele Carraro:** conceptualization (equal), investigation (equal), methodology (supporting), writing – review and editing (equal). **Paolo Cherubini:** conceptualization (equal), investigation (equal), writing – review and editing (equal). **Marco Conedera:** conceptualization (equal), investigation (equal), methodology (equal), writing – review and editing (equal). **Erika Gobet:** conceptualization (equal), investigation (equal), writing – review and editing (equal). **Maria‐Chiara Manetti:** conceptualization (equal), investigation (equal), methodology (equal), writing – review and editing (equal). **Gianluigi Mazza:** investigation (equal), methodology (equal), writing – review and editing (equal). **Christoph Schwörer:** conceptualization (equal), investigation (equal), methodology (equal), writing – review and editing (equal). **Christoph Sperisen:** conceptualization (equal), investigation (equal), methodology (equal), writing – review and editing (equal). **Nadir Alvarez:** conceptualization (equal), investigation (equal), methodology (equal), supervision (equal), writing – review and editing (equal). **Felix Gugerli:** conceptualization (lead), investigation (equal), methodology (equal), supervision (equal), writing – review and editing (equal). **Willy Tinner:** conceptualization (lead), investigation (lead), methodology (equal), project administration (lead), resources (lead), supervision (lead), writing – review and editing (lead).

## Conflicts of Interest

The authors declare no conflicts of interest.

## Supporting information


Appendix S1.

Appendix S2.

Appendix S3.

## Data Availability

The data that support the findings of this study are available in Appendix [Link], [Link] of this article.
