# Supplementary material for: Genetic Differentiation of Abies alba Outside Its Main Range Under Warm Meso‐ and Sub‐Mediterranean Conditions in Italy and Switzerland
Source: Ecol Evol. 2025 Feb 2;15(2):e70909. doi: 10.1002/ece3.70909 (PMC11787904; doi:10.1002/ece3.70909)
Supplement: Supplementary file 1 — Appendix S1. [file ECE3-15-e70909-s003.docx]

**SUPPORTING INFORMATION**

**Genetic differentiation of *Abies alba* outside its main range under warm meso- and sub-Mediterranean conditions in Italy and Switzerland**

Sevil Coşgun, Jérémy Gauthier, Giuliano Bonanomi, Gabriele Carraro, Paolo Cherubini, Marco Conedera, Erika Gobet, Maria-Chiara Manetti, Gianluigi Mazza, Christoph Schwörer, Christoph Sperisen, Nadir Alvarez, Felix Gugerli & Willy Tinner

**Appendix** **1:** Information on DIYABC-RF (Approximate Bayesian Computation with Random Forest) analyses

Analyses of the demographic history was performed in a multi-step procedure due to the complex post-glacial recolonization history of *Abies alba* in Europe. Several scenarios were shaped over different areas based on population structure, evolutionary history and palaeoecological findings in the literature. After determining the best scenario over an area, new scenarios were tested for the next place, keeping the scenarios selected by random forest algorithms as constant. For the populations for which we do not have genetic samples in areas known to be refugia based on fossil evidence, “ghost populations” were added to scenarios. For preliminary scenario analyses, 5,000 simulations were run per scenario. Following each run, best scenarios were selected through random forest of 500 trees (see linear discriminant analysis plots and posterior probabilities) to be used in the further analyses.

1. **Prior scenario testing (with less populations)**

**Switzerland-origin**

1. Eastern/Central Switzerland was recolonized from the ghost refugia, Western Switzerland was recolonized from Eastern/Central Switzerland

2. Eastern/Central Switzerland was recolonized from Western Switzerland and Central Apennines, Western Switzerland was recolonized from the ghost refugia

3. Eastern/Central Switzerland was recolonized from Western Switzerland and Balkans, Western Switzerland was recolonized from the ghost refugia

4. Eastern/Central Switzerland was recolonized from Central Apennines, Western Switzerland was recolonized from Eastern/Central Switzerland

5. Eastern/Central Switzerland was recolonized from Western Switzerland, Western Switzerland was recolonized from the ghost refugia

6. Eastern/Central Switzerland was recolonized from Western Switzerland, Western Switzerland was recolonized from Central Apennines

7. Eastern/Central Switzerland and Western Switzerland were recolonized from the ghost refugia


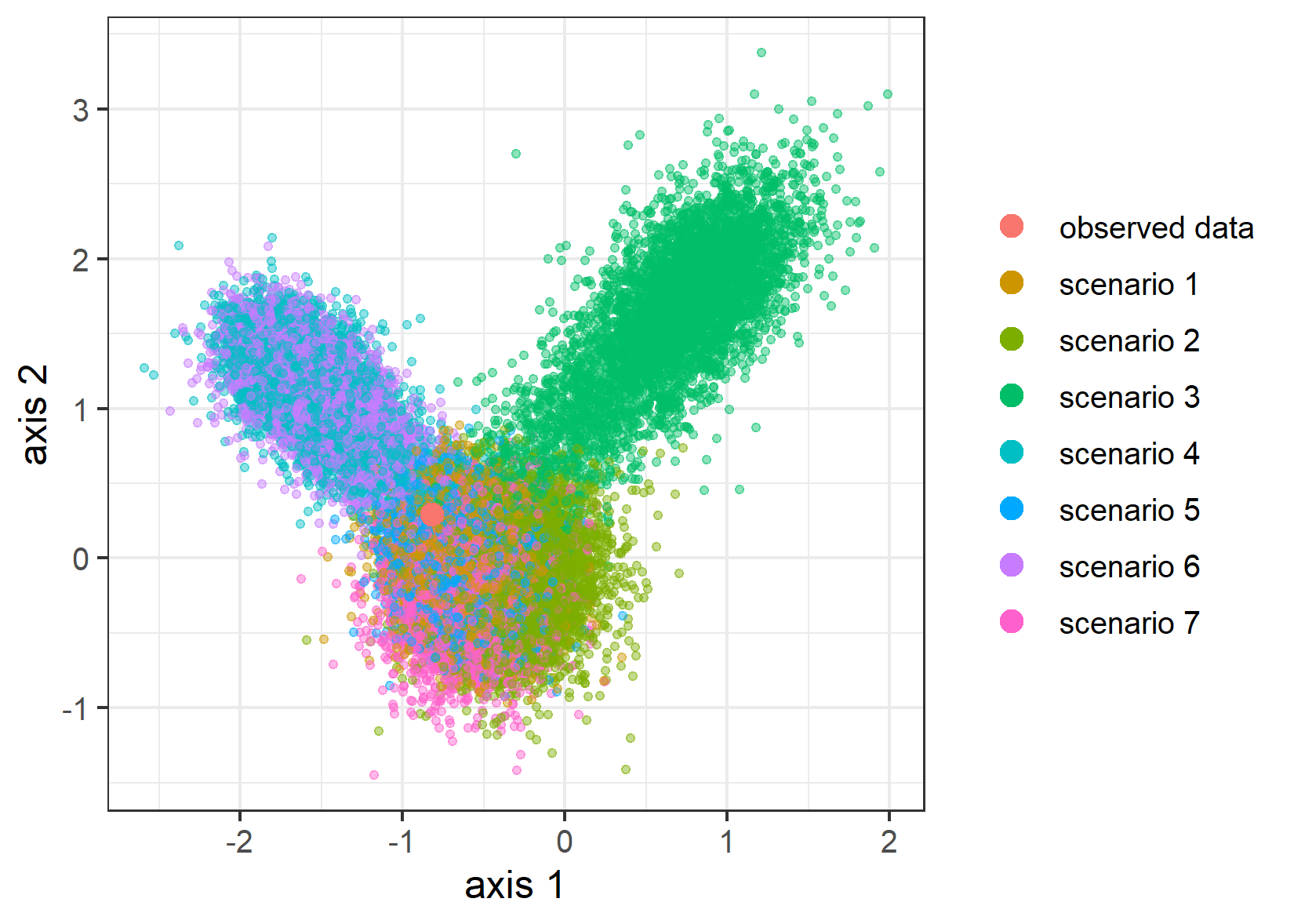


|  | Votes | Posterior probability |
| --- | --- | --- |
| Scenario 1 | 0.244 |  |
| Scenario 2 | 0.072 |  |
| Scenario 3 | 0.102 |  |
| Scenario 4 | 0.052 |  |
| Scenario 5 | 0.338 | 0.566 |
| Scenario 6 | 0.074 |  |
| Scenario 7 | 0.118 |  |

Chosen: Scenario 5

1. **Scenario testing with European set**

**France / Tuscany**

Base scenario: recolonization of Bavaria from Central Apennines, Central/Eastern Switzerland from Western Switzerland and Ticino, Tuscany from Central Apennines and the ghost population from Northern Italy

1. France was recolonized from the ghost population in Northern Italy - Tuscany was recolonized from Central Apennines and the ghost population in Northern Italy
2. France was recolonized from the ghost population in Northern Italy and Pyrenees -Tuscany was recolonized from Central Apennines and the ghost population in Northern Italy
3. France was recolonized from the ghost population in Northern Italy - Tuscany was recolonized from Central Apennines
4. France was recolonized from the ghost population in Northern Italy and Pyrenees Tuscany was recolonized from Central Apennines


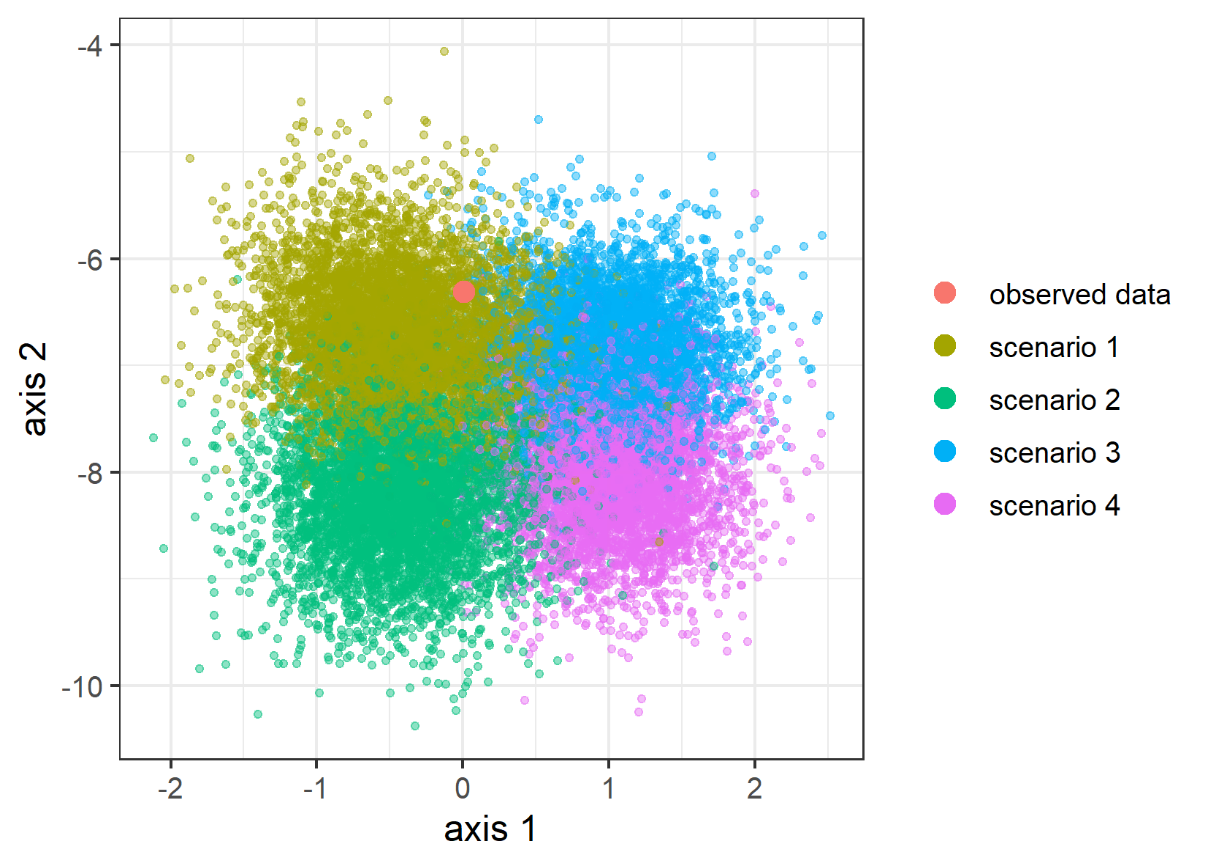


|  |  | Posterior probability |
| --- | --- | --- |
| Scenario 1 | 0.442 | 0.623 |
| Scenario 2 | 0.286 |  |
| Scenario 3 | 0.166 |  |
| Scenario 4 | 0.106 |  |

Chosen: Scenario 1

**Southern Italy**

Base scenario: recolonization of Bavaria from Central Apennines, Central/Eastern Switzerland from Western Switzerland and Ticino, Tuscany from Central Apennines and the ghost population, Pyrenees isolated.

1. Southern Italy was the refugium.
2. Southern Italy was recolonized from the ghost population in Southern Italy & *A. cephalonica.*
3. Southern Italy was recolonized from the ghost population in Southern Italy & Balkan *A. alba.*


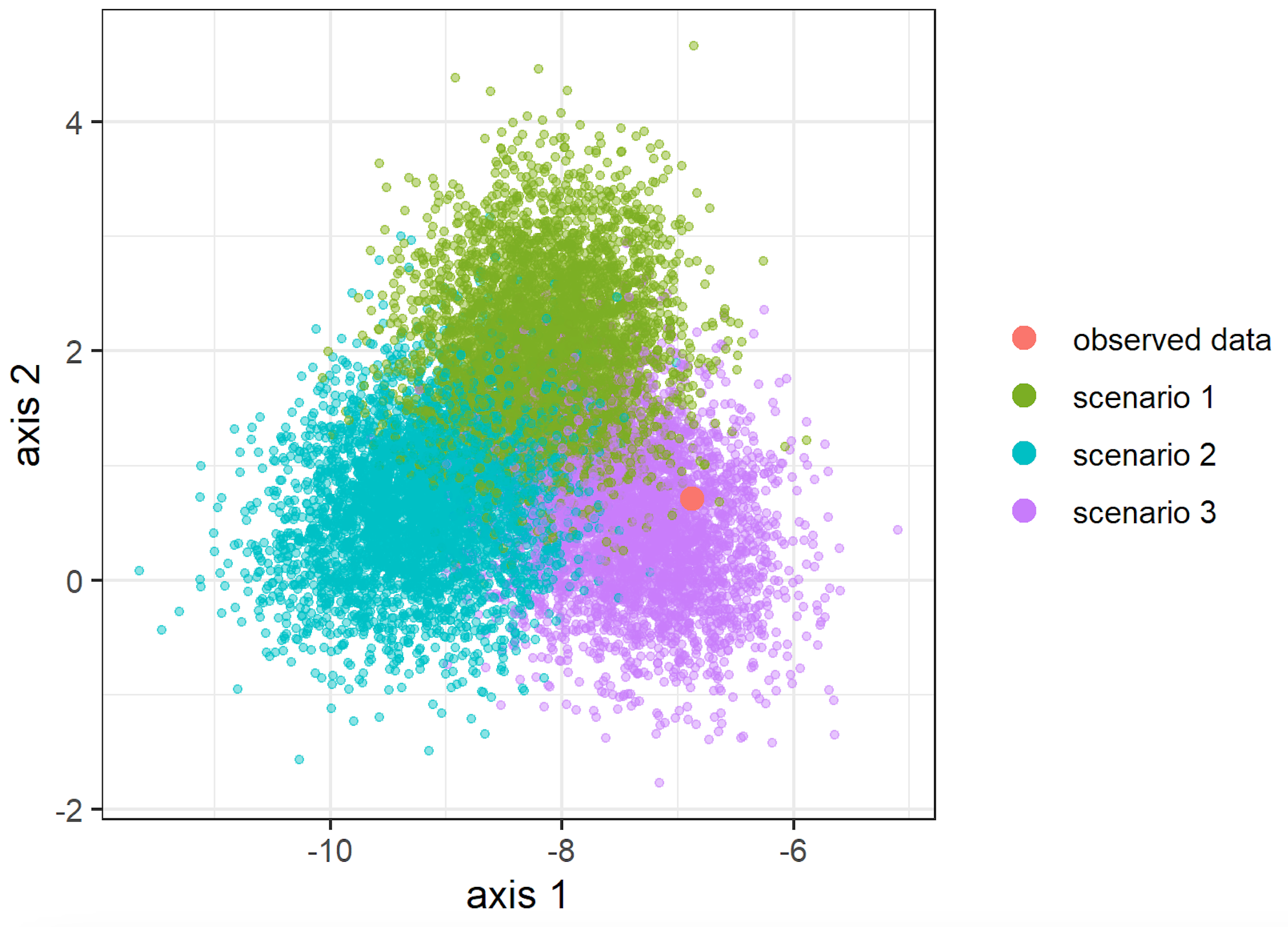


|  |  | Posterior probability |
| --- | --- | --- |
| Scenario 1 | 0.206 |  |
| Scenario 2 | 0.220 |  |
| Scenario 3 | 0.574 | 0.677 |

Chosen: Scenario 3

**Bavaria**

Base scenario: recolonization of Central/Eastern Switzerland from Western Switzerland and Ticino, Tuscany from Central Apennines and the ghost population, Pyrenees isolated.

1. Bavaria was recolonized from Central Apennines
2. Bavaria was recolonized from Central Apennines & Central/Eastern Switzerland

**
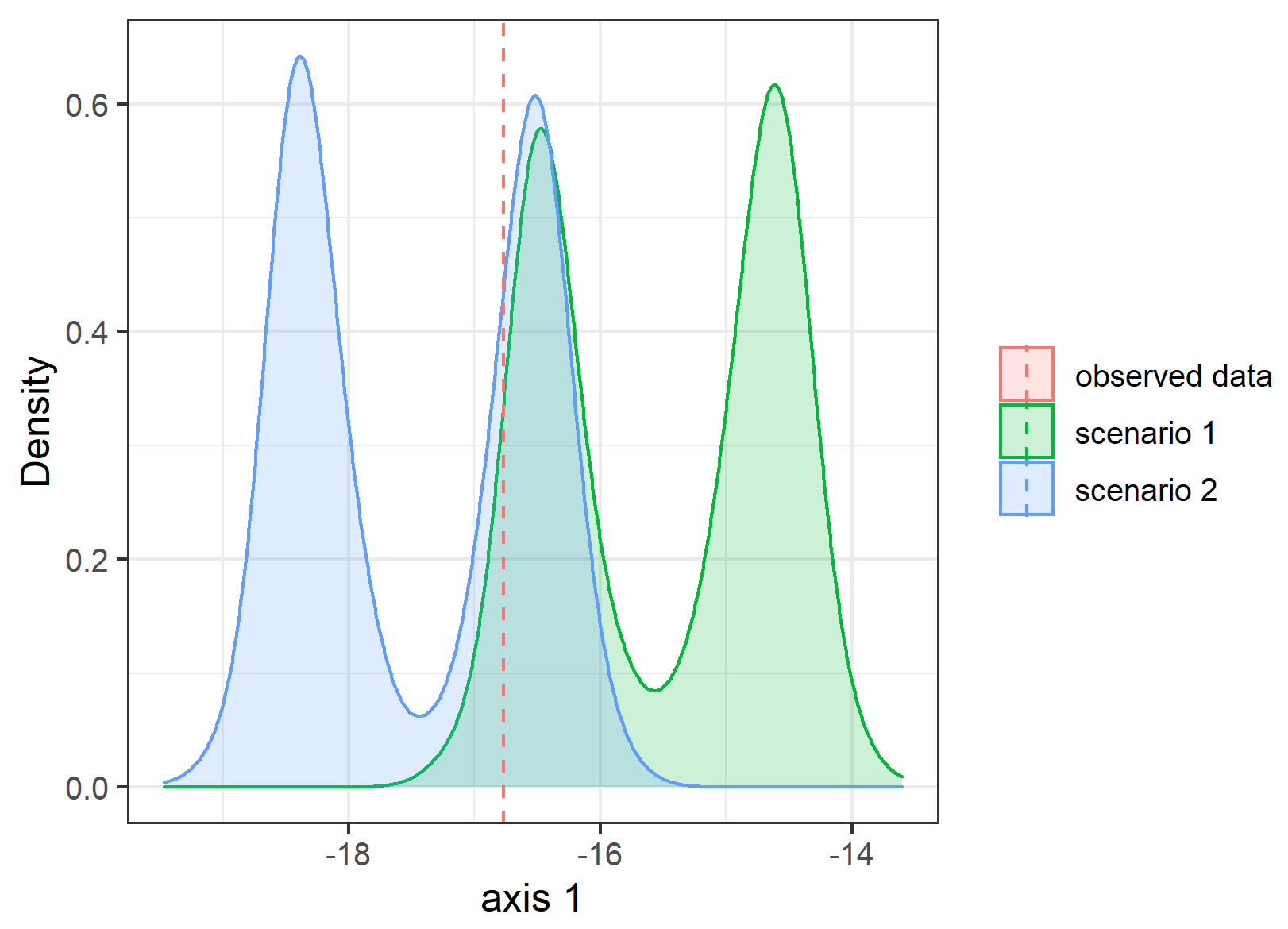
**

|  |  | Posterior probability |
| --- | --- | --- |
| Scenario 1 | 0.116 |  |
| Scenario 2 | 0.884 | 0.878 |

Chosen: Scenario 2

**Central/Eastern Switzerland**

Base scenario: recolonization of Bavaria from Central Apennines and Central CH, Tuscany from Central Apennines and the ghost population in Northern Italy, France from the ghost population, Southern Italy from the ghost population and Balkan *A.alba*

1. Central/Eastern Switzerland was recolonized from Western Switzerland and Ticino, Ticino and Western Switzerland were recolonized from the ghost population.
2. Central/Eastern Switzerland was recolonized from Western Switzerland and Ticino, Ticino was recolonized from the ghost, Western Switzerland was recolonized from the ghost and France.
3. Central/Eastern Switzerland was recolonized from Western Switzerland, Balkan and Ticino, Ticino and Western Switzerland were recolonized from the ghost.
4. Central/Eastern Switzerland was recolonized from Western Switzerland, Balkan and Ticino, Ticino was recolonized from the ghost, Western Switzerland from the ghost and France.
5. Central/Eastern Switzerland was recolonized from Western Switzerland, Balkan and Ticino, Western Switzerland was recolonized from the ghost, Ticino was recolonized from the ghost and Central Italy.
6. Central/Eastern Switzerland was recolonized from Western Switzerland, Balkan and Ticino, Western Switzerland was recolonized from the ghost and France, Ticino was recolonized from the ghost and Central Italy.


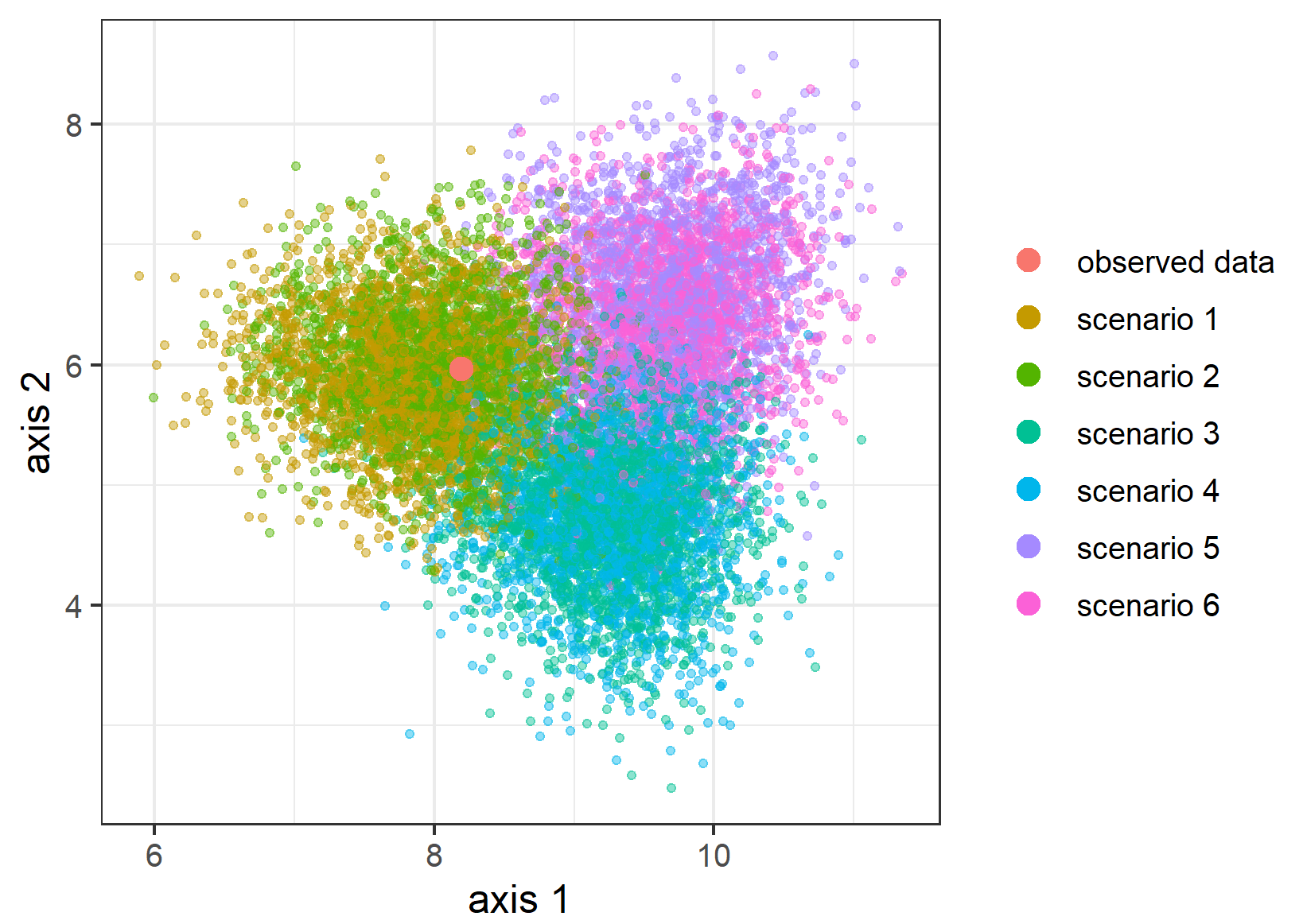


|  |  | Posterior probability |
| --- | --- | --- |
| Scenario 1 | 0.338 | 0.543 |
| Scenario 2 | 0.306 |  |
| Scenario 3 | 0.094 |  |
| Scenario 4 | 0.098 |  |
| Scenario 5 | 0.076 |  |
| Scenario 6 | 0.088 |  |

Chosen: Scenario 1

As seen in the resulting table, Scenario 1 is selected by RF, but with a low posterior probability. Besides, the resulting LDA plot shows an overlap of Scenarios 1 and 2. To look further, we checked the LDA3 and LDA4.


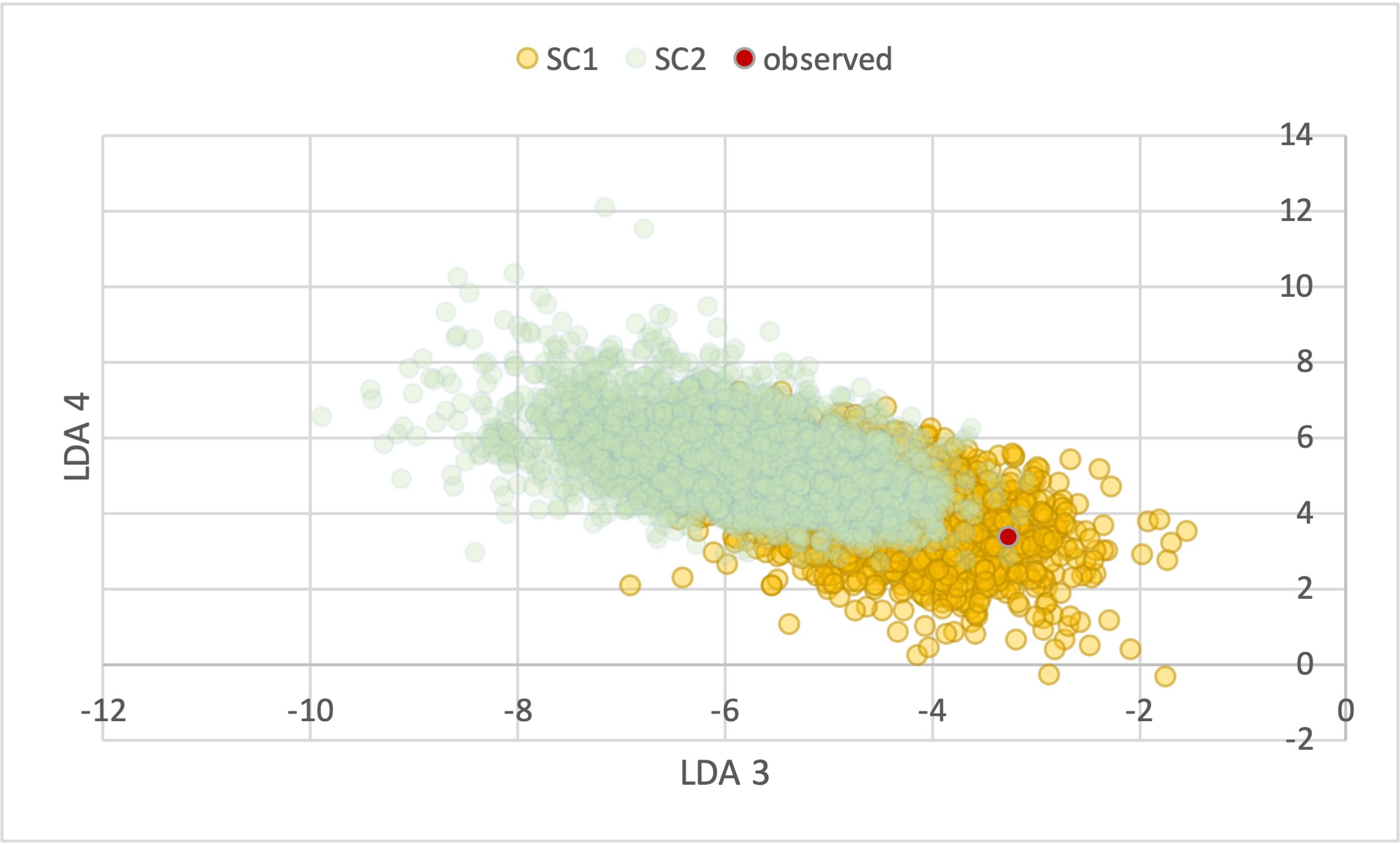


Following the selection of Scenario 1, we further tested the LDA1 and LDA2 of observed value within the normal distribution of Scenario 1.

| LDA1: p= 4.373e-12 | LDA2: p= 8.397e-11 |
| --- | --- |
| 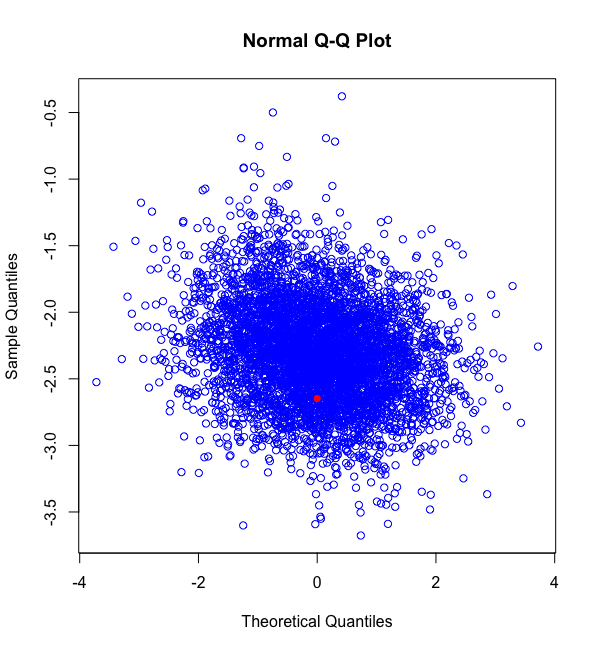 | 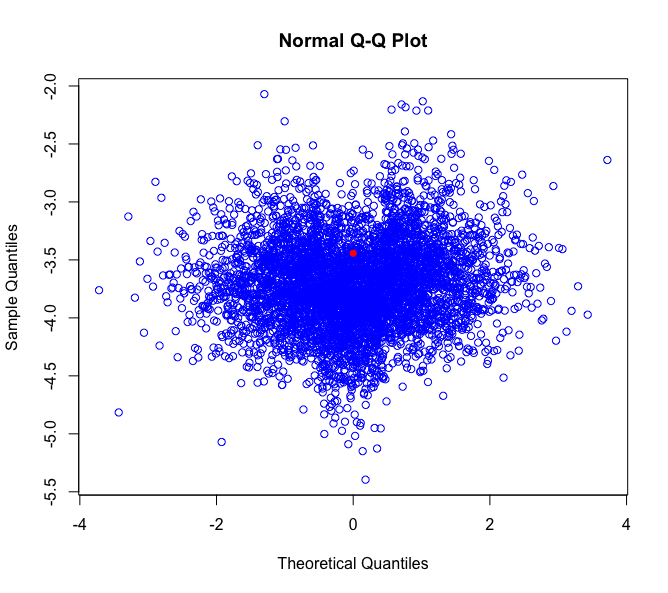 |
|  |  |

1. **Parameter estimation**

**
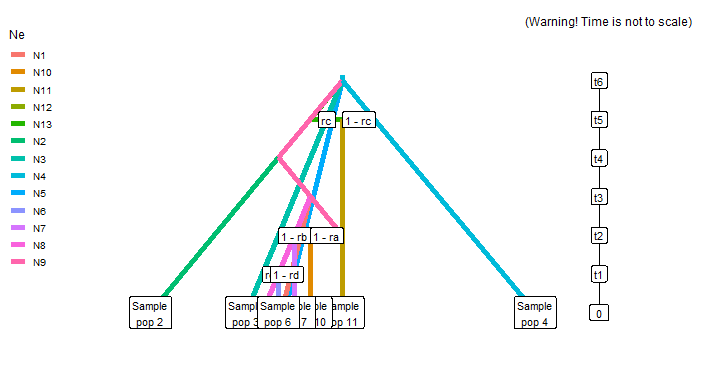
**

After defining the final scenario, parameters were selected with 25,000 simulations followed by a random forest of 500 trees. Priors and estimated values for parameters are shown below.

**Priors for parameter estimation**

|  | **Distribution** | **Minimum** | **Maximum** |
| --- | --- | --- | --- |
| **Priors** |  |  |  |
| N1 | uniform | 10 | 500000 |
| N2 | uniform | 10 | 500000 |
| N3 | uniform | 10 | 500000 |
| N4 | uniform | 10 | 500000 |
| N5 | uniform | 10 | 500000 |
| N6 | uniform | 10 | 500000 |
| N7 | uniform | 10 | 500000 |
| N8 | uniform | 10 | 500000 |
| N9 | uniform | 10 | 500000 |
| N10 | uniform | 10 | 500000 |
| N11 | uniform | 10 | 500000 |
| N12 | uniform | 10 | 500000 |
| N13 | uniform | 10 | 500000 |
| Ne | uniform | 10 | 500000 |
| t1 | uniform | 10 | 250 |
| t2 | uniform | 10 | 250 |
| t3 | uniform | 250 | 20000 |
| t4 | uniform | 10 | 20000 |
| t5 | uniform | 10 | 20000 |
| t6 | uniform | 10 | 50000 |
| rd | uniform | 0.01 | 0.99 |
| rb | uniform | 0.01 | 0.99 |
| ra | uniform | 0.01 | 0.99 |
| rc | uniform | 0.01 | 0.99 |
| **Conditions** |  |  |  |
| t1<t2 |  |  |  |
| t3<t6 |  |  |  |
| t4<t6 |  |  |  |
| t5<t6 |  |  |  |

**Estimated parameters**

| **Parameter** | **Estimated value** | **95% CI** | |
| --- | --- | --- | --- |
| N1 (France) | 47696 | 12035 | 92473 |
| N2 (Pyrenees) | 25718 | 7744 | 36193 |
| N3 (Central Apennines) | 214344 | 103612 | 402003 |
| N4 (Balkan) | 221567 | 11003 | 394542 |
| N5 (Greece) | 59344 | 31336 | 90359 |
| N6 (Bavaria) | 35705 | 89 | 350271 |
| N7 (Central/Eastern Switzerland) | 164020 | 5258 | 457725 |
| N8 (Western Switzerland) | 358016 | 159461 | 491692 |
| N9 (Northern ghost refugium) | 213159 | 79623 | 414105 |
| N10 (Tuscany) | 212365 | 5760 | 470148 |
| N11 (Southern Italy) | 215211 | 33089 | 472776 |
| N12 (Ticino) | 242682 | 85375 | 431371 |
| N13 (Southern ghost refugium) | 229823 | 28249 | 467474 |
| t1 (Bavaria) | 115 | 23 | 218 |
| t2 (Tuscany and Central/Eastern Switzerland) | 173 | 68 | 245 |
| t3 (France, Western Switzerland, and Ticino) | 12385 | 5892 | 18876 |
| t4 (Pyrenees) | 11309 | 3058 | 18781 |
| t5 (Southern Italy) | 575200 | 100950 | 947650 |
| t6 (all lineages from a common ancestor) | 1863700 | 1140450 | 2445000 |
