# Supplementary material for: Genetic Differentiation of Abies alba Outside Its Main Range Under Warm Meso‐ and Sub‐Mediterranean Conditions in Italy and Switzerland
Source: Ecol Evol. 2025 Feb 2;15(2):e70909. doi: 10.1002/ece3.70909 (PMC11787904; doi:10.1002/ece3.70909)
Supplement: Supplementary file 2 — Appendix S2. [file ECE3-15-e70909-s001.docx]

**SUPPORTING INFORMATION**

**Genetic differentiation of *Abies alba* outside its main range under warm meso- and sub-Mediterranean conditions in Italy and Switzerland**

Sevil Coşgun, Jérémy Gauthier, Giuliano Bonanomi, Gabriele Carraro, Paolo Cherubini, Marco Conedera, Erika Gobet, Maria-Chiara Manetti, Gianluigi Mazza, Christoph Schwörer, Christoph Sperisen, Nadir Alvarez, Felix Gugerli & Willy Tinner

**Supplementary material** **1:** Information on sample sources and sampling locations and statistical analyses

Table S1: Sampling locations of *Abies alba* populations compiled from the literature and employed in the European set for this study.

| Population | Country | Coordinates (°N, °E) | References |
| --- | --- | --- | --- |
| VEN | France | 44.17511, 5.2437 | Brousseau et al. (2016) |
| LUR | France | 44.11422, 5.83912 | Brousseau et al. (2016) |
| ISS | France | 44.0242, 6.46244 | Brousseau et al. (2016) |
| VES | France | 43.97074, 7.36577 | Brousseau et al. (2016) |
| PYR | France | 42.855, -0.457778 | Brousseau et al. (2016) |
| VDC | Italy | 42.70347, 13.37576 | Brousseau et al. (2016) |
| CDA | Italy | 42.66772, 13.42677 | Brousseau et al. (2016) |
| ROM2 | Romania | 45.4411, 24.6947 | Brousseau et al. (2016) |
| BUL | Bulgaria | 41.843055, 23.3852 | Brousseau et al. (2016) |
| GRE | Greece | 37.68333, 22.20639 | Brousseau et al. (2016) |
| BAV | Germany | 48.975, 13.4 | Heer et al. (2018) |
| BEI | Switzerland | 47.23, 8.318 | Csilléry et al. (2020) |
| BON | Switzerland | 46.324, 9.541 | Csilléry et al. (2020) |
| BRS | Switzerland | 46.595, 6.175 | Csilléry et al. (2020) |
| COR | Switzerland | 47.162, 7.055 | Csilléry et al. (2020) |
| GRB | Switzerland | 47.334, 9.114 | Csilléry et al. (2020) |
| GRY | Switzerland | 46.299, 7.091 | Csilléry et al. (2020) |
| JEZ | Switzerland | 46.921, 9.7 | Csilléry et al. (2020) |
| LUT | Switzerland | 46.634, 7.952 | Csilléry et al. (2020) |
| MGY | Switzerland | 46.095, 7.1 | Csilléry et al. (2020) |
| MUO | Switzerland | 46.991, 8.708 | Csilléry et al. (2020) |
| NFS | Switzerland | 47.09, 8.997 | Csilléry et al. (2020) |
| POS | Switzerland | 46.27, 10.082 | Csilléry et al. (2020) |
| PRA | Switzerland | 46.479, 8.75 | Csilléry et al. (2020) |
| SIG | Switzerland | 46.891, 7.761 | Csilléry et al. (2020) |
| SIR | Switzerland | 46.28, 7.56 | Csilléry et al. (2020) |
| TSC | Switzerland | 46.938, 10.481 | Csilléry et al. (2020) |
| VAZ | Switzerland | 46.639, 7.002 | Csilléry et al. (2020) |
| VRG | Switzerland | 46.237, 8.53 | Csilléry et al. (2020) |
| VWD | Switzerland | 46.273, 7.884 | Csilléry et al. (2020) |

Table S2: Climatic and vegetation information for the *Abies alba* stands sampled for this study.

| **Population name** | **Mean**  **annual temperature**  **(°C)** | **Annual precipitation**  **(mm)** | **July temperature**  **(°C)** | **Other species** |
| --- | --- | --- | --- | --- |
| Bern | 7.75 | 1407 | 16.95 | *Fagus sylvatica, Picea abies* |
| Graubünden | 4.35 | 867 | 14.45 | *P. abies, Sorbus aucuparia* |
| Ticino1 | 11.85 | 1715 | 21.95 | *Castanea sativa, Quercus petraea, Tilia cordata, Fraxinus excelsior, Ilex aquifolium, Acer campestre, Rubus* sp. |
| Ticino2 | 10.75 | 1783 | 20.75 | *P. abies, C. sativa, Acer pseudoplatanus, F. sylvatica, Taxus baccata, Calluna vulgaris, Juniperus communis, Erica carnea, T. cordata, F. excelsior, I. aquifolium, A. campestre, Rubus* sp. |
| Ticino3 | 8.95 | 1809 | 18.85 | *C. sativa, F. sylvatica, T. cordata, Q. petraea* |
| Ticino4 | 6.45 | 2004 | 16.25 | *P. abies, F. sylvatica, Larix decidua* |
| Ticino5 | 2.45 | 2543 | 12.15 | *P. abies, F. sylvatica, L. decidua* |
| Ticino6 | 2.45 | 2543 | 12.15 | *P. abies, F. sylvatica, L. decidua* |
| Ticino7 | 5.45 | 2603 | 15.35 | *P. abies, F. sylvatica, L. decidua* |
| Ticino8 | 3.65 | 1608 | 13.95 | *P. sylvestris, P. abies, Corylus avellana, C. sativa* |
| Tuscany1 | 14.55 | 847 | 24.05 | *Fraxinus ornus, C. sativa, Quercus cerris, Quercus ilex, Carpinus betulus, Ilex aquifolium* |
| Tuscany2 | 12.65 | 1003 | 22.65 | *F. ornus, C. sativa, Q. cerris, Q. ilex, C. betulus* |
| Tuscany3 | 11.15 | 1063 | 20.65 | *C. sativa, A. pseudoplatanus, F. sylvatica, Prunus avium, C. betulus, Ulmus glabra*, *I. aquifolium* |
| Tuscany4 | 12.55 | 907 | 22.35 | *F. ornus, C. sativa, Q. cerris, Q. ilex, C. betulus, P. avium* |
| Tuscany5 | 6.15 | 831 | 15.15 | *F. sylvatica, P. abies* |
| Molise1 | 7.95  10.55 | 1113  1117 | 17.15 | *P. abies, F. sylvatica, I. aquifolium* |
| Molise2 | 10.55 | 1117 | 19.85 | *F. sylvatica, Q. cerris, C. betulus* |
| Basilicata1 | 12.65 | 706 | 22.25 | *A. pseudoplatanus*, *C. sativa, L. decidua, P. abies, F. sylvatica* |
| Basilicata2 | 9.55 | 853 | 18.55 | *F. sylvatica, Q. cerris, O. carpinifolia, I. aquifolium, A. campestre* |
| Basilicata3 | 7.95 | 1058 | 16.45 | *F. sylvatica, Q. cerris, Malus sylvestris* |
| Campania | 8.35 | 1072 | 17.35 | *I. aquifolium, C. avellana, F. sylvatica, Ostrya carpinifolia, Acer* sp.*, Tilia platyphyllos* |
| Calabria | 12.65 | 1197 | 20.55 | *F. sylvatica, C. sativa, Alnus glutinosa* |

*Climatic data was obtained through CHELSA V 2.1 for 1981-2010 (Karger et al., 2017, 2018).

Table S3: Observed heterozygosity, expected heterozygosity, and allelic richness for the *Abies alba* stands sampled for this study.

| Population name | He | Ho | Ar |
| --- | --- | --- | --- |
| Bern | 0.269 | 0.255 | 1.752 |
| Graubünden | 0.252 | 0.261 | 1.705 |
| Ticino2 | 0.266 | 0.25 | 1.736 |
| Ticino3 | 0.265 | 0.262 | 1.74 |
| Ticino4 | 0.266 | 0.266 | 1.761 |
| Ticino5 | 0.268 | 0.281 | 1.764 |
| Ticino6 | 0.272 | 0.281 | 1.739 |
| Ticino7 | 0.263 | 0.261 | 1.736 |
| Ticino8 | 0.249 | 0.261 | 1.709 |
| Tuscany1 | 0.26 | 0.262 | 1.699 |
| Tuscany2 | 0.258 | 0.254 | 1.704 |
| Tuscany3 | 0.258 | 0.25 | 1.72 |
| Tuscany4 | 0.23 | 0.24 | 1.6 |
| Tuscany5 | 0.26 | 0.262 | 1.732 |
| Molise1 | 0.175 | 0.195 | 1.48 |
| Molise2 | 0.175 | 0.174 | 1.493 |
| Basilicata1 | 0.18 | 0.172 | 1.524 |
| Basilicata2 | 0.188 | 0.192 | 1.51 |
| Basilicata3 | 0.178 | 0.177 | 1.514 |
| Campania | 0.187 | 0.191 | 1.524 |
| Calabria | 0.179 | 0.176 | 1.509 |
| Black Forest | 0.254 | 0.246 | 1.739 |
| Romania | 0.222 | 0.199 | 1.586 |


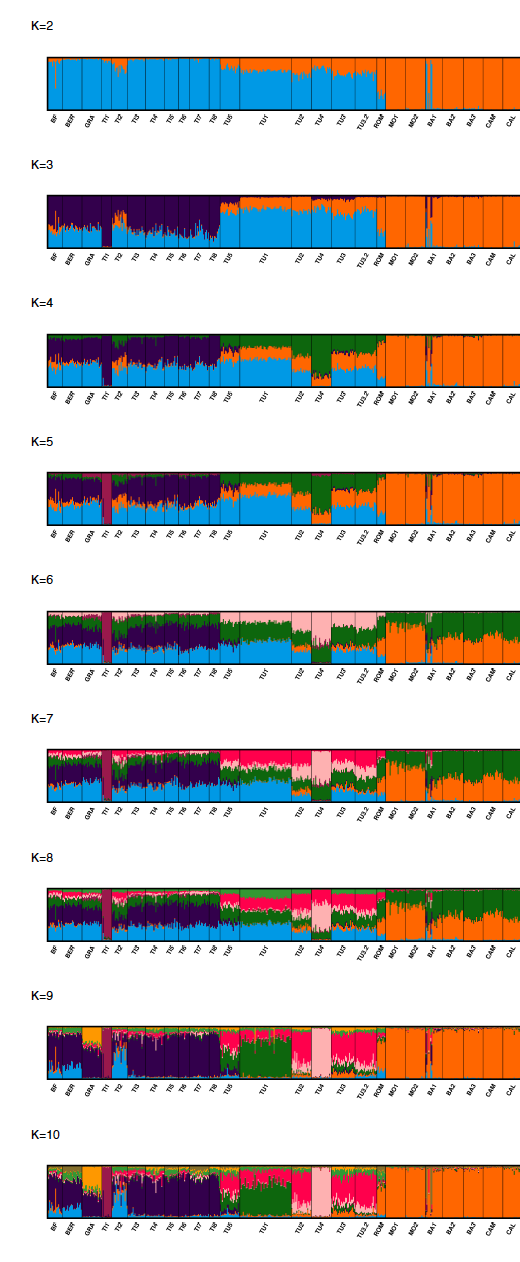


Figure S1: Genetic structure, based on assignment probabilities to K=1-10 clusters, of *Abies alba* stands sampled for this study including outlier populations. Each horizontal bar represents one individual, population codes on the x axis as in Table 1.


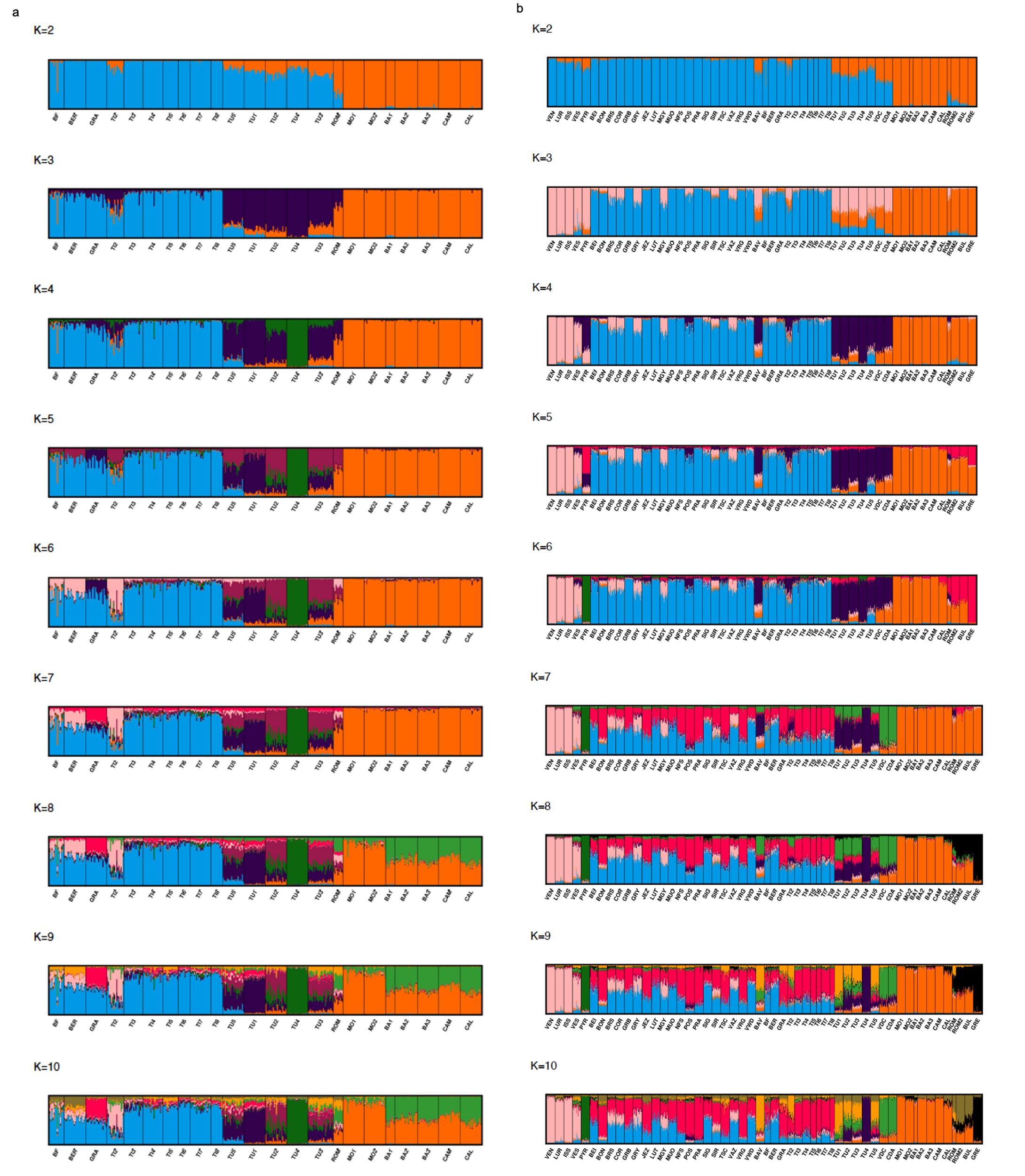


Figure S2: Genetic structure, based on assignment probabilities to K=1-10 clusters, for the samples collected in this study (174 SNPs genotyped; a) and of *Abies alba* stands for the European set (112 SNPs commonly genotyped including the literature data; b). Each horizontal bar represents one individual, population codes on the x axis as in Tables 1 and S1.


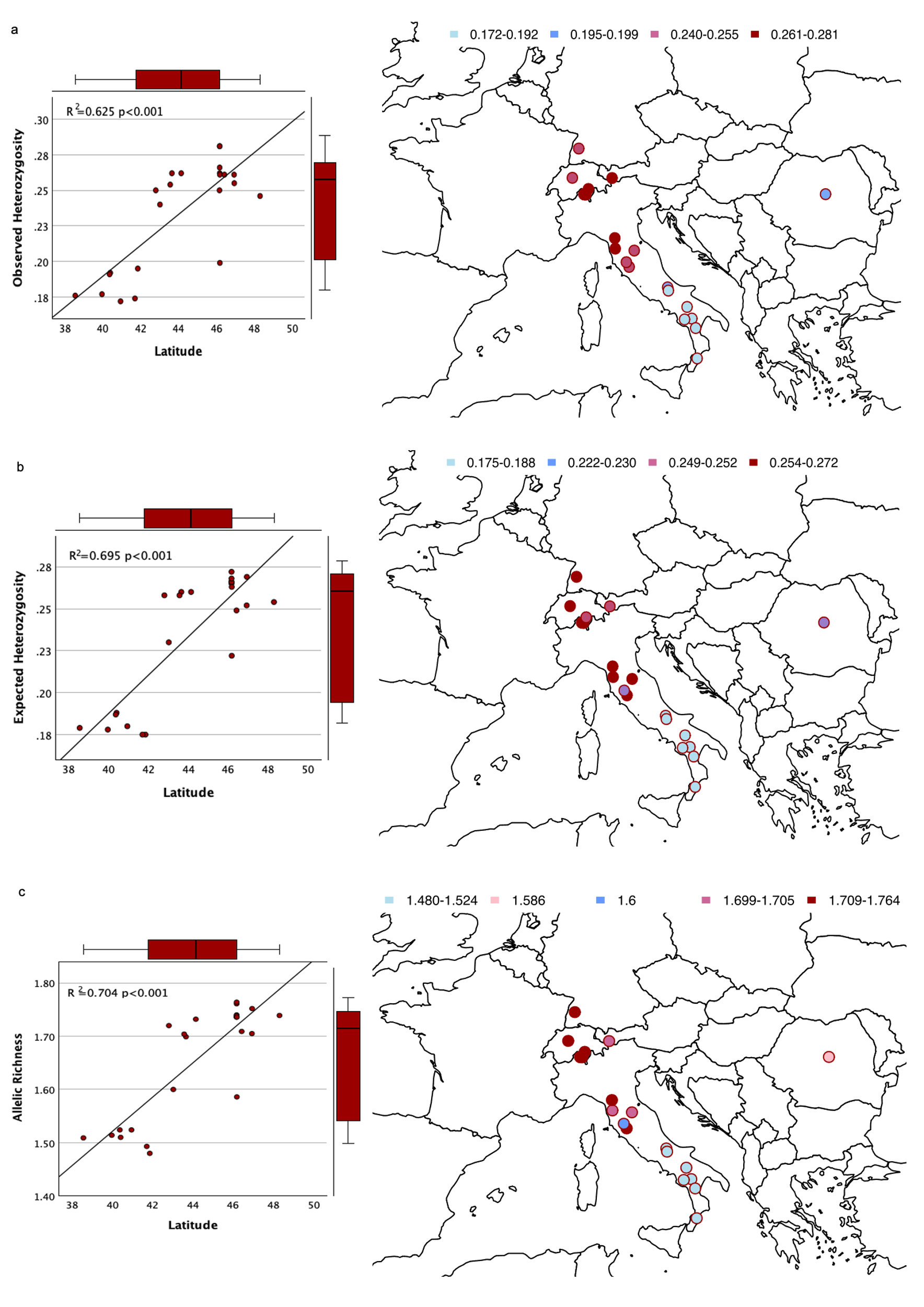


Figure S3: Observed heterozygosity (Ho; a), expected heterozygosity (He; b), and allelic richness (Ar; c) for the samples of *Abies alba* collected for this study (174 SNPs genotyped) with linear regression of diversity measures on latitude; R^2^ and levels of significance are given in the insets.


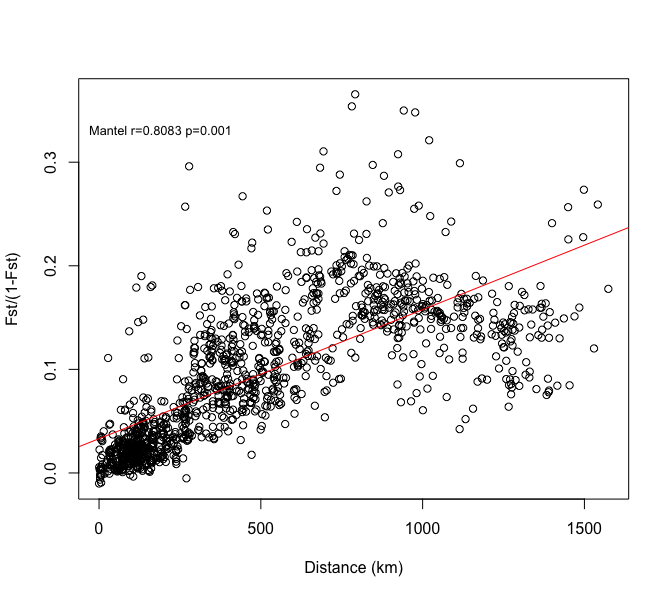


Figure S4: Isolation by distance for the European set (excluding populations from Greece and Pyrenees) of *Abies alba,* using linearized pairwise genetic (Fst) and geographic distances (km) in a Mantel matrix correlation based on Spearman's Rho.

**SNP selection and genotyping**

A subset of 174 putatively neutral SNPs were selected from Roschanski et al. (2016) from those with relatively high Fst values by checking that they were not paralogs (Supplementary material 2-SNPs for KASP genotyping). Subsequently, STRUCTURE analyses (Falush et al., 2003, 2007; Hubisz et al., 2009; Pritchard et al., 2000) were repeated with the dataset and parameters of Csilléry et al. (2020) to ensure having a similar clustering with a reduced number of SNPs.

A total of 478 *A. alba* DNA samples were genotyped using KASP arrays by LGC Genomics (Berlin, Germany). We excluded the samples with more than 10% missing data. We then identified outlier loci with BayeScan v.2.1. (20 pilot runs of 5000 iterations each and a burn-in of 50,000, 10 prior odds for the neutral model) (Foll & Gaggiotti, 2008) and Arlequin v3.5 (20000 simulations; Excoffier et al., 2009; Excoffier & Lischer, 2010) and extracted SNPs detected by both approaches prior to further evolutionary and demographic history analyses.

**Population structure**

To have a uniform sample size in the European set with the literature data (Brousseau et al., 2016; Csilléry et al., 2020; Heer et al., 2018) ~20 samples were randomly selected from each site. 100,000 burn-in and 1,000,000 Markov Chain Monte Carlo repetitions were run in the Bayesian assignment in STRUCTURE, choosing the LOCPRIOR model together with the admixture and correlated allele frequencies options. For each K in the range of 1-10, ten iterations were run, and STRUCTURE output was subjected to the greedy and large greedy algorithms in CLUMPP (Jakobsson & Rosenberg, 2007), as implemented in CLUMPAK (Kopelman et al., 2015). To determine the number of genetic clusters that best explained our data, plotting of the LnP(D) (giving the mean and the variance over iterations) and the Evanno method (change in the slope of LnP(D) curve) were employed (Evanno et al., 2005; Janes et al., 2017; Pritchard et al., 2000), as implemented in Structure Harvester v0.6.94 (Earl & vonHoldt, 2012). STRUCTURE results were visualized by Structure selector (Li & Liu, 2018).

LnP(K) and ΔK plots suggest different numbers of clusters (Fig. S5). According to ΔK (Evanno et al., 2005), the best number of clusters could be determined as K=2. However, there are concerns about the tendency of Evanno’s method to define two clusters (for further details, see Janes et al. (2017)). In turn, the LnP(K) plot reached a plateau at K=4.


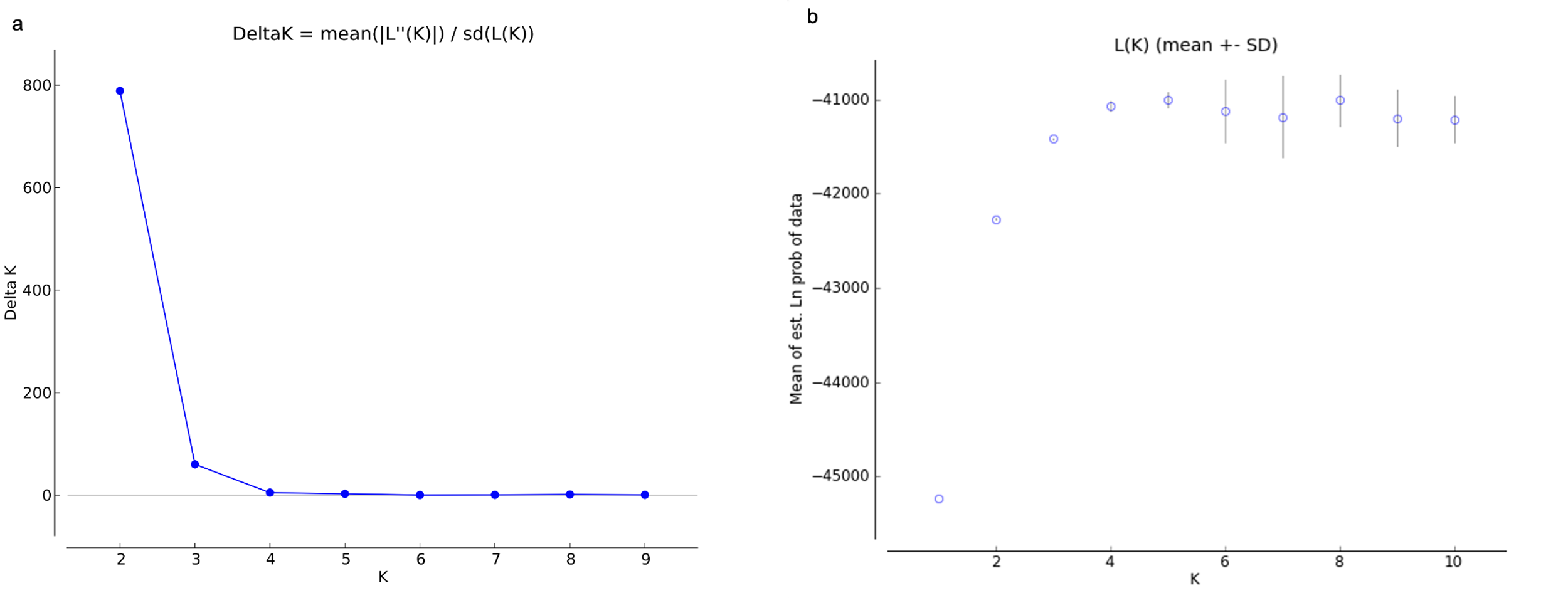


Figure S5: Rate of change of the likelihood (a) and the mean of estimated likelihood (b) as a function of increasing K in the range of 1-10 as determined by Structure Harvester v0.6.94 (Earl & vonHoldt, 2012).

**Evolutionary history**

For analyses on the evolutionary history in European set, we rooted the outgroup *Abies cephalonica* population from Brousseau et al. (2016) and ran TreeMix with 3 iterations for 0 -10 migrations, with no resampling blocks of SNPs . The variance explained by the model was estimated with the ‘get_f()’ of TreeMix implemented in R. The optimal number of migration events is determined using the linear method by the *optM* R package (Fitak, 2021). We then performed the Three-Population Test in TreeMix using the ‘threepop’ function, based on the estimation of f3 as in Reich et al. (2009) and by looking for the Z-scores less than -3, as an indication of historical admixtures.

Representing the optimum number of migrations, change points (where an increasing number of migrations does not produce a significant increase in likelihood) were determined by optM (Fitak, 2021) as 1, 1, 2.98, and 2.93 by simple exponential, non-linear least squares, piecewise linear, and bent cable methods, respectively (Fig. S6). As Fitak (2021) reported that the change points by the simple exponential and non-linear least squares models should not be considered accurate, we kept three migrations (from Pyr to Ves: 36%, within Switzerland, from Rom to Tsc&Gra: 24%). With this model, the maximum likelihood tree with three migrations explained 91.12% of the total variation. However, the presence of residuals above zero indicates that the tree could not wholly explain the evolutionary history of the samples (Fig. S7).


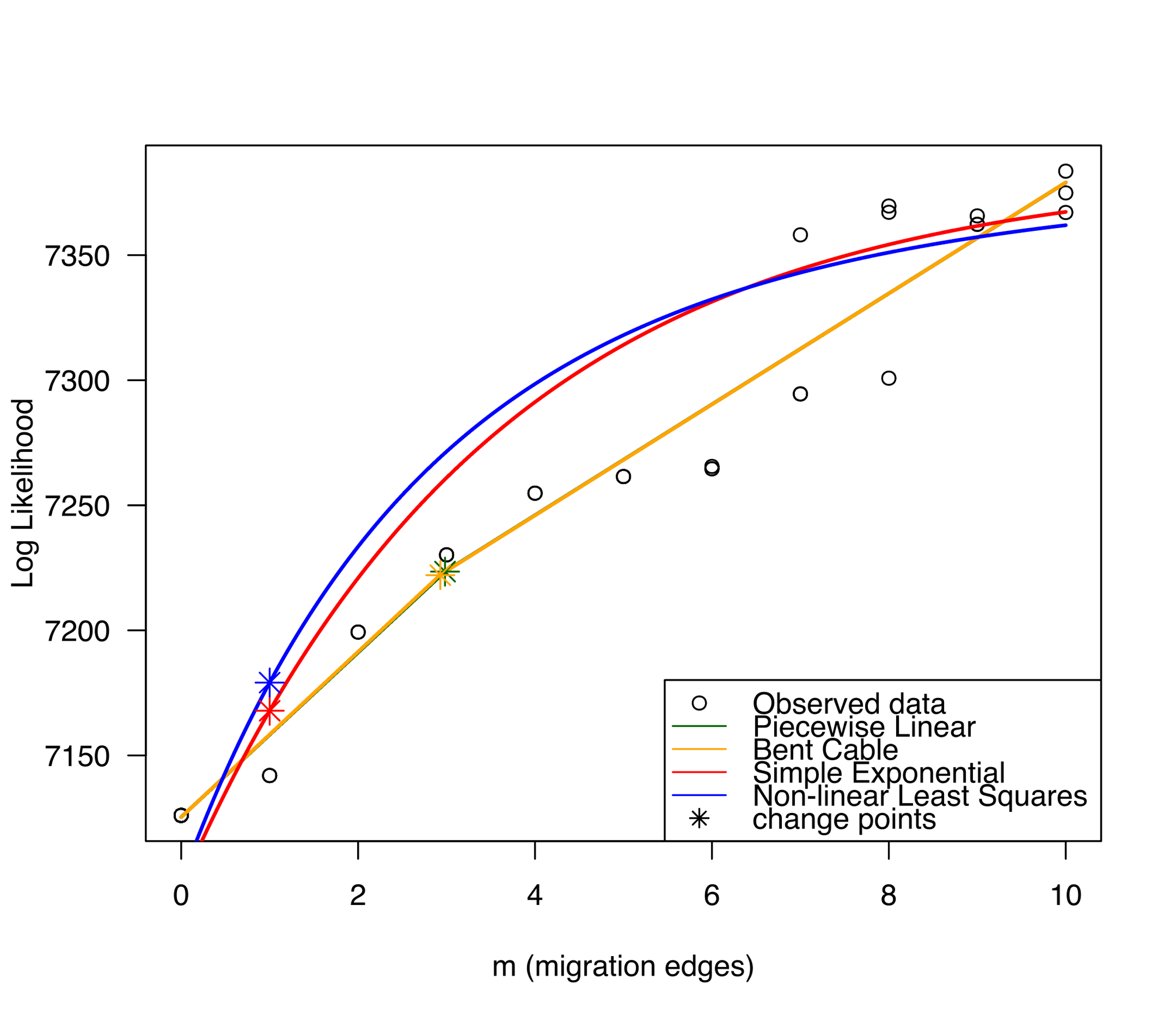


Figure S6: Change points of the likelihood as a function of increasing M in the range of 1-10 using four different ecological threshold models fit as determined by OptM {Fitak, 2021 #222}.


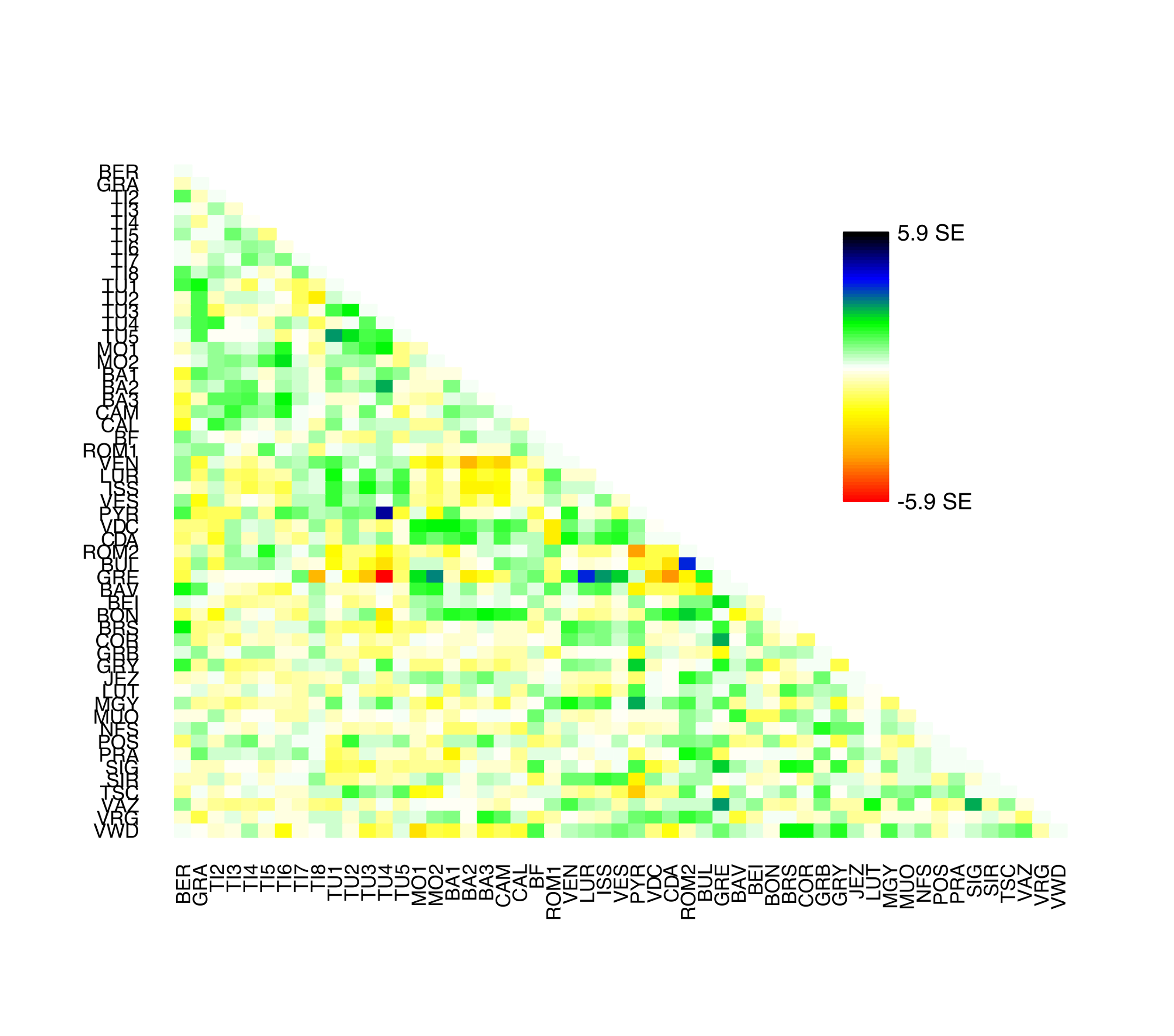


Figure S7: Scaled residuals from the fit of the model with three migrations to the data of *Abies alba*. Positive residuals represent populations that are more closely related to each other than the model and possible candidates for admixture. Population codes along both axes as in Tables 1 and S1.

REFERENCES

Brousseau, L., Postolache, D., Lascoux, M., Drouzas, A. D., Källman, T., Leonarduzzi, C., Liepelt, S., Piotti, A., Popescu, F., Roschanski, A. M., Zhelev, P., Fady, B., & Vendramin, G. G. (2016). Local adaptation in European firs assessed through extensive sampling across altitudinal gradients in Southern Europe. *PLOS ONE, 11*(7), e0158216. <https://doi.org/10.1371/journal.pone.0158216>

Csilléry, K., Ovaskainen, O., Sperisen, C., Buchmann, N., Widmer, A., & Gugerli, F. (2020). Adaptation to local climate in multi-trait space: evidence from silver fir (*Abies alba Mill*.) populations across a heterogeneous environment. *Heredity (Edinb), 124*(1), 77-92. <https://doi.org/10.1038/s41437-019-0240-0>

Earl, D. A., & vonHoldt, B. M. (2012). STRUCTURE HARVESTER: a website and program for visualizing STRUCTURE output and implementing the Evanno method. *Conservation Genetics Resources, 4*(2), 359-361. <https://doi.org/10.1007/s12686-011-9548-7>

Evanno, G., Regnaut, S., & Goudet, J. (2005). Detecting the number of clusters of individuals using the software structure: a simulation study. *Molecular Ecology, 14*(8), 2611-2620. <https://doi.org/10.1111/j.1365-294X.2005.02553.x>

Excoffier, L., Hofer, T., & Foll, M. (2009). Detecting loci under selection in a hierarchically structured population. *Heredity, 103*(4), 285-298. <https://doi.org/10.1038/hdy.2009.74>

Excoffier, L., & Lischer, H. E. L. (2010). Arlequin suite ver 3.5: a new series of programs to perform population genetics analyses under Linux and Windows. *Molecular Ecology Resources, 10*(3), 564-567. <https://doi.org/10.1111/j.1755-0998.2010.02847.x>

Falush, D., Stephens, M., & Pritchard, J. K. (2003). Inference of population structure using multilocus genotype data: linked loci and correlated allele frequencies. *Genetics, 164*(4), 1567-1587. <https://doi.org/10.1093/genetics/164.4.1567>

Falush, D., Stephens, M., & Pritchard, J. K. (2007). Inference of population structure using multilocus genotype data: dominant markers and null alleles. *Molecular Ecology Notes, 7*(4), 574-578. <https://doi.org/10.1111/j.1471-8286.2007.01758.x>

Fitak, R. R. (2021). OptM: estimating the optimal number of migration edges on population trees using Treemix. *Biology Methods and Protocols, 6*(1). <https://doi.org/10.1093/biomethods/bpab017>

Foll, M., & Gaggiotti, O. (2008). A Genome-Scan Method to Identify Selected Loci Appropriate for Both Dominant and Codominant Markers: A Bayesian Perspective. *Genetics, 180*(2), 977-993. <https://doi.org/10.1534/genetics.108.092221>

Heer, K., Behringer, D., Piermattei, A., Bässler, C., Brandl, R., Fady, B., Jehl, H., Liepelt, S., Lorch, S., Piotti, A., Vendramin, G. G., Weller, M., Ziegenhagen, B., Büntgen, U., & Opgenoorth, L. (2018). Linking dendroecology and association genetics in natural populations: Stress responses archived in tree rings associate with SNP genotypes in silver fir (*Abies alba* Mill.). *Molecular Ecology, 27*(6), 1428-1438. <https://doi.org/10.1111/mec.14538>

Hubisz, M. J., Falush, D., Stephens, M., & Pritchard, J. K. (2009). Inferring weak population structure with the assistance of sample group information. *Molecular Ecology Resources, 9*(5), 1322-1332. <https://doi.org/10.1111/j.1755-0998.2009.02591.x>

Jakobsson, M., & Rosenberg, N. A. (2007). CLUMPP: a cluster matching and permutation program for dealing with label switching and multimodality in analysis of population structure. *Bioinformatics, 23*(14), 1801-1806. <https://doi.org/10.1093/bioinformatics/btm233>

Janes, J. K., Miller, J. M., Dupuis, J. R., Malenfant, R. M., Gorrell, J. C., Cullingham, C. I., & Andrew, R. L. (2017). The K = 2 conundrum. *Molecular Ecology, 26*(14), 3594-3602. <https://doi.org/https://doi.org/10.1111/mec.14187>

Karger, D. N., Conrad, O., Böhner, J., Kawohl, T., Kreft, H., Soria-Auza, R. W., Zimmermann, N. E., Linder, H. P., & Kessler, M. (2017). Climatologies at high resolution for the earth’s land surface areas. *Scientific Data, 4*(1), 170122. <https://doi.org/10.1038/sdata.2017.122>

Karger, D. N., Conrad, O., Böhner, J., Kawohl, T., Kreft, H., Soria-Auza, R. W., Zimmermann, N. E., Linder, H. P., & Kessler, M. (2018). *Data from: Climatologies at high resolution for the earth's land surface areas.* <https://doi.org/10.5061/dryad.kd1d4>

Kopelman, N. M., Mayzel, J., Jakobsson, M., Rosenberg, N. A., & Mayrose, I. (2015). Clumpak: a program for identifying clustering modes and packaging population structure inferences across K. *Molecular Ecology Resources, 15*(5), 1179-1191. <https://doi.org/10.1111/1755-0998.12387>

Li, Y. L., & Liu, J. X. (2018). StructureSelector: A web-based software to select and visualize the optimal number of clusters using multiple methods. *Mol Ecol Resour, 18*(1), 176-177. <https://doi.org/10.1111/1755-0998.12719>

Pritchard, J. K., Stephens, M., & Donnelly, P. (2000). Inference of population structure using multilocus genotype data. *Genetics, 155*(2), 945-959. <https://doi.org/10.1093/genetics/155.2.945>

Reich, D., Thangaraj, K., Patterson, N., Price, A. L., & Singh, L. (2009). Reconstructing Indian population history. *Nature, 461*(7263), 489-494. <https://doi.org/10.1038/nature08365>

Roschanski, A. M., Csilléry, K., Liepelt, S., Oddou-Muratorio, S., Ziegenhagen, B., Huard, F., Ullrich, K. K., Postolache, D., Vendramin, G. G., & Fady, B. (2016). Evidence of divergent selection for drought and cold tolerance at landscape and local scales in Abies alba Mill. in the French Mediterranean Alps. *Molecular Ecology, 25*(3), 776-794. <https://doi.org/10.1111/mec.13516>
